# Molecular Dynamics Insights into the Aggregation Behavior of N-Terminal β-Lactoglobulin Peptides

**DOI:** 10.3390/ijms25094660

**Published:** 2024-04-25

**Authors:** Srdjan Pusara

**Affiliations:** Institute of Nanotechnology, Karlsruhe Institute of Technology KIT, Kaiserstraße 12, 76131 Karlsruhe, Germany; srdjan.pusara@partner.kit.edu

**Keywords:** β-lactoglobulin, β-lactoglobulin peptides, peptide aggregation, amyloid aggregation, early-stage amyloid aggregation, molecular dynamics, all-atom molecular dynamics

## Abstract

β-lactoglobulin (BLG) forms amyloid-like aggregates at high temperatures, low pH, and low ionic strengths. At a pH below 2, BLG undergoes hydrolysis into peptides, with N-terminal peptides 1–33 and 1–52 being prone to fibrillization, forming amyloid-like fibrils. Due to their good mechanical properties, BLG amyloids demonstrate great potential for diverse applications, including biosensors, nanocomposites, and catalysts. Consequently, further studies are essential to comprehensively understand the factors governing the formation of BLG amyloid-like morphologies. In this study, all-atom molecular dynamics simulations were employed to explore the aggregation of N-terminal 1–33 and 1–52 BLG peptides under conditions of pH 2 and at 10 mM NaCl concentration. The simulations revealed that the peptides spontaneously assembled into aggregates of varying sizes. The aggregation process was enabled by the low charge of peptides and the presence of hydrophobic residues within them. As the peptides associated into aggregates, there was a concurrent increase in β-sheet structures and the establishment of hydrogen bonds, enhancing the stability of the aggregates. Notably, on average, 1–33 peptides formed larger aggregates compared to their 1–52 counterparts, while the latter exhibited a slightly higher content of β-sheets and higher cluster orderliness. The applied approach facilitated insights into the early stages of amyloid-like aggregation and molecular-level insight into the formation of β-sheets, which serve as nucleation points for further fibril growth.

## 1. Introduction

BLG is the most abundant protein found in the whey fraction of cow’s milk, garnering substantial attention in research due to its importance in the food industry and rich aggregation behavior. BLG consists of a single polypeptide chain containing 162 amino acids, with a spherical structure and comprising nine antiparallel β-sheets and a single α-helix. It demonstrates the capacity to aggregate, under diverse conditions, into amyloid-like or random aggregates [1,2]. The characteristics and sizes of the BLG aggregates are influenced by the solution conditions, such as pH, ionic strength and temperature of the solution, as well as solvent type, stirring speed, incubation time, or protein concentration [3]. While misfolded amyloid aggregates are usually associated with serious human disorders like Alzheimer’s disease, type-II diabetes, and Creutzfeldt–Jakob disease [4], it was recognized that amyloid aggregates can form from all proteins under certain conditions, including BLG and food proteins. Amyloids can also serve functional roles in certain organisms, strengthening bacterial biofilms and composing structural components in fish and insect eggshells [1]. Ultimately, amyloid aggregates can find diverse applications in material science, and, to enhance their effectiveness, it is preferable to utilize proteins abundantly present in common dietary sources, such as BLG.

At a pH close to the isoelectric point (∼pH 5.2), BLG aggregates into compact, monodisperse, randomly connected spherical aggregates (~150 nm) [5], while at a neutral pH, electrostatic repulsion leads to unbranched, short (<100 nm) and worm-like aggregates with a diameter of 6 ± 1 nm [5,6]. The amyloid nature of these aggregates was confirmed using a Thioflavin T (ThT) assay, a common indicator of amyloid aggregation, as reported in the study by Da Silva Pinto et al. [6].

However, at a lower pH, BLG aggregates into smaller amyloid(-like) aggregates, resulting in typical semi-flexible fibrils. At a pH from 3 to 3.5 and elevated temperatures > 70 °C, BLG partially unfolds and it assembles into randomly associated amyloid-like aggregates that are thin (1–3 nm) and short (50–200 nm), typically arranged into worm-like aggregates [5,7,8]. Under pH < 3, the aggregation of intact BLG becomes unfavorable due to strong electrostatic repulsion; however, the acidic environment induces hydrolysis into shorter peptides, which serve as building blocks for the formation of amyloid fibrils. It was demonstrated that, at pH 2, BLG undergoes hydrolysis (typically several hours at 80–90 °C), yielding peptides within the range of 2000 to 8000 Da [7,9,10]. Some of the hydrolyzed peptides exhibit an increased tendency to form fibrils, ultimately aggregating into fibrillar structures, while others remain unaggregated. This gives a typical yield of 40–50% when compared to the total protein mass. Notably, the N-terminal sequences 1–32, 1–33, 1–52, and 1–53 peptides exhibit a strong affinity for fibrillization, exclusively found within fibrils, while partial tendencies toward fibrillization are observed in peptides such as 12–33, 99–129, 138–162, and so forth, which can be present in both fibrils and non-aggregated material [1,9,10]. The increased propensity for association of these particular peptide sequences is primarily ascribed to their heightened hydrophobicity, as well as their ability to form β-sheets, and low peptide charge. The amyloid fibrils obtained by this heat treatment at pH 2 and low ionic strength (circa 10 mM NaCl) are straight, thin (2.6 to 4 nm), long (1 to 10 µm), and well-ordered [11]. Intermolecular β-sheets hold together these peptide building blocks in these fibrils, running perpendicular to the fibril axis. Their unique dimensions make them appealing for applications like food product thickeners. In contrast, under higher ionic strengths, electrostatic interactions are screened, resulting in shorter fibrils organized into morphologies resembling a warm-like structure.

Amyloid aggregates derived from BLG or whey protein isolate (WPI) have demonstrated capabilities as effective emulsifiers [12], gelators [13], encapsulating agents [14], and carriers for substances like curcumin [15] and iron [16]. Furthermore, films derived from BLG amyloid aggregates exhibit remarkable strength, comparable in elastic modulus to keratin and collagen. Amyloid aggregates of BLG offer diverse potential uses, including applications in biosensors, nanocomposites, and catalysts [17]. While the safety of engineered aggregates for human consumption is still debated and needs resolution, the impressive qualities of natural amyloid structures inspire the creation of engineered amyloid aggregates from food proteins.

The assembly of BLG nanofibrils begins with nucleation, followed by fibril growth, culminating in termination. Nucleation involves reversible protein/peptide assembly, which becomes irreversible upon reaching a critical nucleus size [2,18,19]. Fibril growth occurs through the linear addition of building blocks to a nucleus, yielding a highly ordered fibril structure. Ultimately, termination occurs when building blocks are depleted [1,2,19]. The fibril formation typically reaches a stationary phase within 24 h of thermal treatment or longer [1].

While extensive research efforts have focused on the physicochemical properties of BLG fibrils and the solution conditions that lead to the formation of diverse aggregate structures [7], our understanding of their all-atom structural composition remains limited. This challenge arises from the difficulty in employing techniques capable of revealing their detailed atomic composition; therefore, many studies have primarily relied on transmission electron microscopy (TEM) to gather information about aggregate morphology. The first-ever reported partial description of the atomistic structure of BLG fibrils was undertaken by Gowda et al. [20]. They investigated a synthetic peptide fragment consisting of residues 11–20 of the protein (which is a central region of 1–33 sequence thathas high affinity for fibrillization), using it to facilitate fibril growth on fibril seeds formed through BLG hydrolysis. Employing a range of techniques, including nuclear magnetic resonance (NMR) measurements with site-specific labeling, they confirmed the presence of parallel β-sheet structures and deduced inter-residue contacts. However, the NMR data did not conclusively determine the inter-sheet packing. As a result, the study turned to molecular dynamics simulations to explore various packing modes and compare them with the NMR data [20]. Other than this, no other computational or molecular dynamics studies were employed to study aggregation of BLG proteins or BLG-derived peptides into amyloid fibrils. Some studies have reported using kinetic theory to model BLG fibril growth and compare it with experimental data: however, the structural resolution of fibrils and their constituent building blocks was not considered [21].

**Figure 1:**
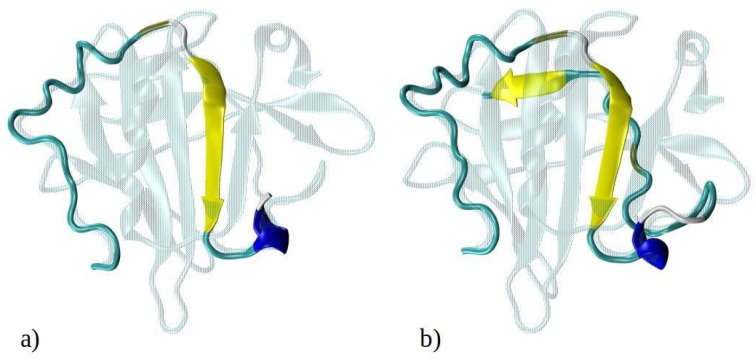

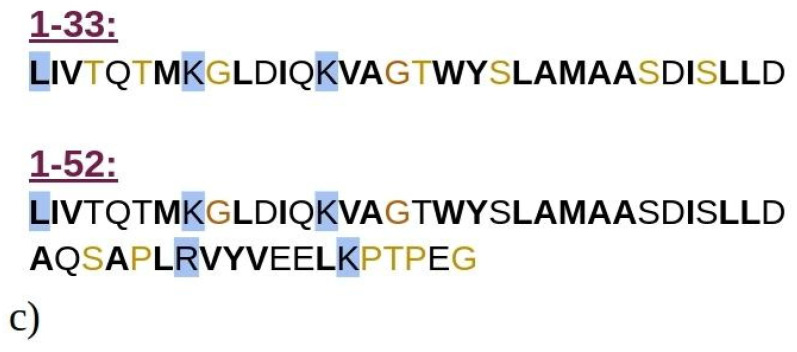
Graphical representation of the crystal structure of beta-lactoglobulin protein with the emphasized structures of (**a**) 1–33 and (**b**) 1–52 N-terminal peptides (i.e., peptides consisting of the first 33 and 52 amino acids of BLG), simulated using MD. The peptides are color-coded to represent their secondary structure: yellow for β-sheets, blue for helices, and cyan for random coils. For illustration purposes, the entire BLG protein structure is shown in transparent cyan, superimposed onto the peptides to indicate their precise locations within the protein. (**c**) Schematic representation of amino acids composing the 1–33 and 1–52 peptides. Amino acids are represented by standard one-letter codes. Hydrophobic residues are marked by bold black font, neutral residues by bold orange font, and hydrophilic residues by unbolded black font. Positively charged residues are highlighted by a light blue background, while all other residues are uncharged.

Computational techniques, particularly molecular dynamics (MD) simulations, offer a robust approach to investigate the early self-assembly phase of protein or peptide aggregation, including amyloid aggregation. This phase is challenging to capture experimentally due to rapid aggregation and transient (short-living) intermediate oligomers. While MD simulations have been extensively used to study the aggregation of amyloid-prone peptides associated with various conditions, including Alzheimer’s disease [22,23,24,25,26,27,28,29], in-depth computational studies on amyloid aggregation of BLG or its peptide fragments remain scarce. In this study, we employed MD simulations to explore the aggregation of N-terminal 1–33 and 1–52 peptides from BLG, i.e., known building blocks of BLG amyloid fibrils at low pH and low ionic strengths [9]. The N-terminal peptides 1–33 and 1–52 are shown in Figure 1, superimposed onto the entire crystal structure of the BLG protein. Both 1–33 and 1–52 peptides are mostly composed of hydrophobic or neutral residues (Figure 1c), highlighting their potential role in amyloid aggregation, which will be investigated in this manuscript. Our simulations enabled us to observe the initial stages of spontaneous amyloid fibril formation and track the transformation of early disordered aggregates into ordered structures rich in β-sheets, a critical step in the development of highly ordered fibrillar structures.

## 2. Results and Discussion

As key constituents of BLG fibrils under conditions of low pH and low ionic strength, the 1–33 and 1–52 peptides were chosen for this study. The objective of the present work was to investigate the early stages of peptide aggregation to understand the associated structural changes throughout the aggregation process and molecular bases of stable β-sheets-based fibril formation. 

### 2.1. Early Step Aggregation: Size Distribution Analysis

From MD simulations of single 1–33 and 1–52 peptides (see Figures S1 and S2), high chain flexibility at simulated conditions was observed. In summary, 1–33 peptides predominantly exhibited random coil conformations, whereas 1–52 peptides assumed more folded structures, progressing in simulations to form intramolecular β-sheets (refer to Figure S2 for snapshots). To maintain the structural diversity of peptides under simulated conditions, twenty random peptide conformations were used to generate unbiased starting configurations for simulating the aggregation process. The starting snapshots of MD simulations are shown in Figure S3.

**Figure 2:**
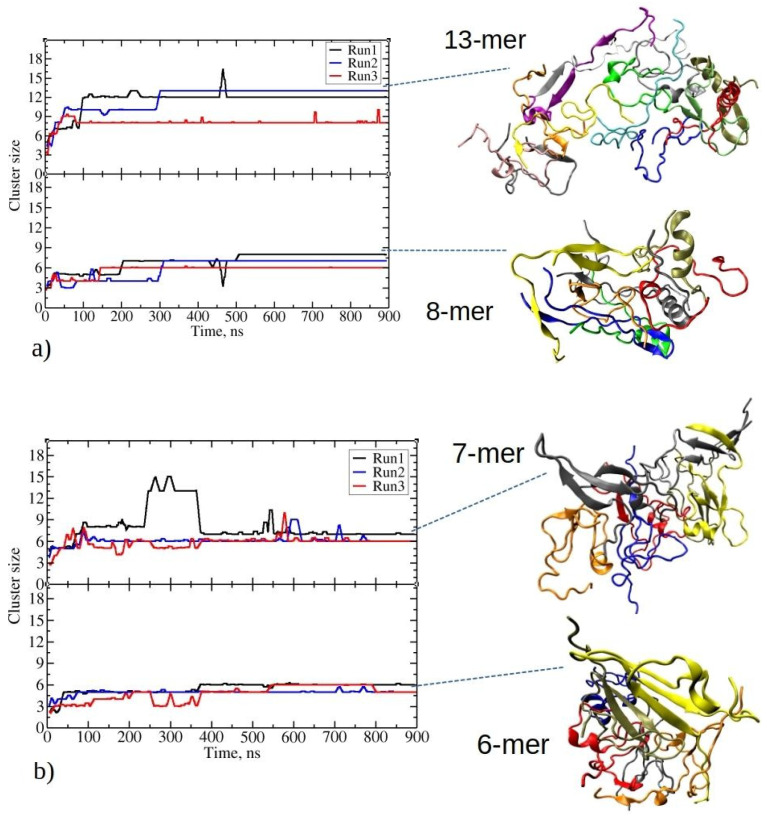
Time evolution for the two largest clusters in MD simulations involving twenty peptides of (**a**) 1–33 and (**b**) 1–52. The top and bottom panels of each plot represent the evolution of the largest and second-largest aggregates observed in the corresponding MD run. On the right side, representative snapshots of clusters taken from the end of the MD trajectory are presented. For 1–33: an aggregate comprising thirteen and eight peptides from Run2 and Run1, respectively. For 1–52: an aggregate comprising seven and six peptides from Run1. Each peptide chain in the aggregate (cluster) is colored differently.

Despite both peptide types having a slightly positive total charge, they rapidly aggregated, forming clusters in the early simulation stages without any notable lag phase. The low net charge of peptides has been demonstrated to expedite the aggregation rate of amyloid peptides overall [30,31]. Furthermore, hydrophobicity plays an even more significant role in promoting amyloid fibril formation. Figure 2 illustrates the temporal evolution of the two largest clusters observed during three independent MD runs for each peptide type. Insets on the right side depict examples of the clusters formed that persisted throughout the final stages of simulations. During MD simulations with 20 copies of 1–33 and 1–52 peptides, they predominantly maintained folded conformations, although they were more elongated than in single-peptide simulations (Figure S4).

At the beginning of MD runs of 1–33 peptides, the size of the largest aggregate gradually increased, giving rise to intermediate aggregates comprising seven to eight peptides (Figure 2a). This phase was followed by a notably rapid growth to the maximum aggregate size within the subsequent 100 to 300 ns. Afterward, the size of the largest aggregate generally remained stable during the simulations, marked by minor fluctuations, with one to three peptide chains intermittently associating with or dissociating from the largest aggregate. The maximum aggregate sizes at the end of three independent simulations were twelve, thirteen, and eight, respectively. The inset in Figure 2a illustrates the observed clusters that formed, showcasing various structural motifs, including β-sheets, helices, or random coils. The secondary structure is described in detail in the next section. Stable fibril formation, as detected experimentally, should be facilitated by further structural rearrangements, surpassing the microsecond MD simulations analyzed in the present paper [1].

In the lower panel in Figure 2a, the time evolution of the second-largest cluster of 1–33 is depicted. The second-largest aggregates consist of eight, seven, and six peptides, respectively. Thus, in two out of three simulation runs of 1–33 peptides, all peptides are exclusively found within one of the two largest aggregates during the final stages of the simulations, with no other aggregates present. The final distribution of peptide aggregates likely results from competitive growth among smaller clusters, culminating in the formation of two predominant peptide aggregates.

Similarly, 1–52 peptides undergo early-stage aggregation during MD simulations, achieving the largest aggregate sizes within the initial 100 ns. From the inset structures on Figure 2b, the initial formation of β-strands is noticeable for these peptides. The largest aggregates maintain stable size throughout simulations, except in Run1.

In the first MD run of 1–52, the largest aggregate grows into a cluster with eight peptides at 90 ns, persisting for the next 140 ns before evolving into a larger aggregate of 13 peptides (Figure 2b). This larger aggregate maintains stability for an additional 120 ns, with occasional acceptance and release of new peptides (i.e., 2–4 peptides at 260 ns and 300 ns). By 365 ns, the largest aggregate loses five peptides, holding at seven peptides until the simulation’s end. Notably, data on Figure 2 employ a 100-step running average (within the last 1 ns), omitting short-lived transitions in the plots. Conversely, in the other two MD runs with twenty copies of 1–52 peptides, aggregation results in stable largest clusters of sizes six and six, which persist until the end of the simulation. Therefore, in comparison to the shorter 1–33 peptides, 1–52 peptides form smaller aggregates, resulting in the size of the largest aggregates of seven, six, and six peptides at the end of each run, respectively. The second-largest aggregates contain six, five, and five peptides. The formation of smaller aggregates in this case appears to be influenced by longer, more folded peptide chains compared to 1–33 peptides (refer to Figure S2). The presence of pronounced intramolecular interactions limits their ability to establish intermolecular connections. These findings suggest that, due to their larger size, 1–52 peptides require more time to reorganize and establish larger, longer-lasting (i.e., stable) aggregates. 

**Figure 3:**
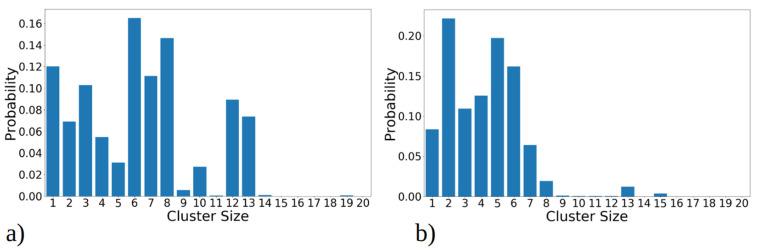

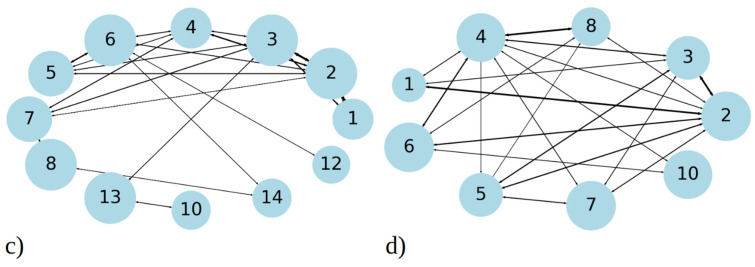
Cluster size distribution observed during simulation of 20 copies of (**a**) 1–33 and (**b**) 1–52 peptides. Transition matrix illustrating the 40 most frequent transitions between cluster sizes for (**c**) 1–33 and (**d**) 1–52 peptide simulations. The data presented in these plots include statistics from all three MD runs combined, for 1–33 and 1–52 peptides, respectively. The size of the circle is proportional to the total number of transitions from/into that state, while the thickness of the line is proportional to the number of events between two states. Both graphs represent the data gained from 2.7 µs simulation, i.e., the sum of three MD runs.

The total cluster size distribution over all MD runs, revealing how frequently each cluster was observed throughout MD simulations, is illustrated in Figure 3a,b. This analysis uncovers distinct trends for the 1–33 and 1–52 peptides. Specifically, for 1–33 peptides, as shown on Figure 3a, the presence of both large clusters (i.e., with twelve or thirteen peptides) and intermediate-sized clusters (comprising seven, eight, or ten peptides) is observed. In contrast, 1–52 peptides predominantly form smaller clusters, typically comprising two to five peptides (Figure 3b). Medium-sized, stable aggregates (i.e., with six, seven, or eight peptides) occur less frequently. Larger clusters form only transiently during simulations, likely through the fusion of smaller clusters, but they lack long-term stability. 

In addition to analyzing aggregate size distribution, we gained insights into aggregate dynamics from transition networks, illustrated in Figure 3c,d. A transition network summarizes the evolution of aggregates, i.e., the transition pathways from the one cluster size to the other during MD simulations. For 1–33 peptides, three main categories of transitions are revealed (Figure 3c). Firstly, single peptides frequently join into two- and three-membered clusters; therefore, the number of these events is high and the circle, representing it, is large compared to other ones. These assemblies proceed with some merging into larger clusters, for example to four-membered or seven-membered clusters. Secondly, transitions occur between intermediate seven- and eight-membered clusters, which are the second-largest long-living clusters we observed in MD runs (Figure 2a). Along with these transformations, the dissociation onto smaller clusters (four- and five-membered) takes place. Lastly, the largest and most stable clusters (12 and 13- membered) exhibit frequent transitions among themselves, occasionally accepting smaller aggregates to grow into larger clusters (14- and 17-membered). Full transition networks in Figure S5a show that 19- and 20-membered clusters also occasionally form by coalescence of smaller clusters, however they are short-lived and, thus, not visible in Figure 3c.

In contrast, MD simulations of 1–52 peptides showed, on the one hand, a similar pattern, where numerous transitions between one-, two-, and three-membered clusters (Figure 3d) occur. However, these simulations feature significantly more transitions between intermediate clusters, particularly those with four to eight members, representing the largest stable aggregates during MD runs (as shown on Figure 2b). The full transition map in Figure S5b shows occasional transitions to larger aggregates such as to 12-, 13-, 14-, 15-, and 20-membered clusters; however, these transitions are less frequent. Overall, 1–52 peptides mostly aggregate into intermediate size clusters, which occasionally accept or release single peptides or smaller aggregates, consistent with data in Figure 2b. 

### 2.2. Solvent-Accessible Surface Area

**Figure 4:**
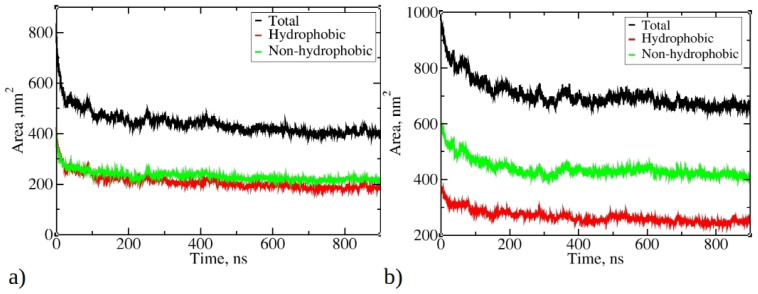
Change of the total solvent-accessible surface (SASA) during simulations of 20 copies of (**a**) 1–33 and (**b**) 1–52 peptides. Only data from Run 1 are presented for each peptide, as main trends remained consistent across multiple runs.

Figure 4 illustrates the change in total solvent-accessible surface area (SASA) during MD simulations of 1–33 and 1–52 peptides, as well as SASA originating from hydrophobic and non-hydrophobic residues. As the simulations progress, there is a significant reduction in SASA, aligning with the progressive aggregation of peptides. The final SASA for 1–33 and 1–52 peptides stabilizes at around 400 nm² and 660 nm², respectively, significantly lower than their initial values (810 nm² and 970 nm²). Notably, the SASA contribution from hydrophobic residues is smaller than that from hydrophilic and neutral residues. The SASA ratio of hydrophobic to non-hydrophobic residues does not align with peptide compositions. In 1–33 peptides, where approximately 55.3% of atoms belong to hydrophobic residues, their SASA is smaller than the SASA of other residue types.

Conversely, in 1–52 peptides, with around 46.2% of atoms belonging to hydrophobic residues, the contribution of hydrophobic residues to SASA is significantly smaller compared to their proportion within the peptide. This observation suggests that during aggregation, hydrophobic residues tend to aggregate and, therefore, minimize contact with water, while hydrophilic residues preferentially occupy the outer surface of aggregates, interacting favorably with water molecules. The reduction in SASA is primarily driven by the aggregation process facilitated by hydrophobic residues, minimizing their exposure to water. This underscores the role of the hydrophobic effect as hydrophobic residues from different peptides interact, reducing overall SASA and favoring aggregation. 

### 2.3. Secondary Structure

Amyloid fibrils typically feature parallel arrangement of peptides forming beta sheets perpendicularly to the fibril axis. Fibrils formed from BLG are 1 to 10 µm long and were reported to be formed over 24 h [1]. This time exceeds the time scale of MD simulations; therefore, we did not aim to simulate fibril formation, but to understand early steps of assembly, which precede further nucleation and fibril growth. Thus, the change of secondary structure of 1–33 and 1–52 peptides during MD simulations was investigated.

**Figure 5:**
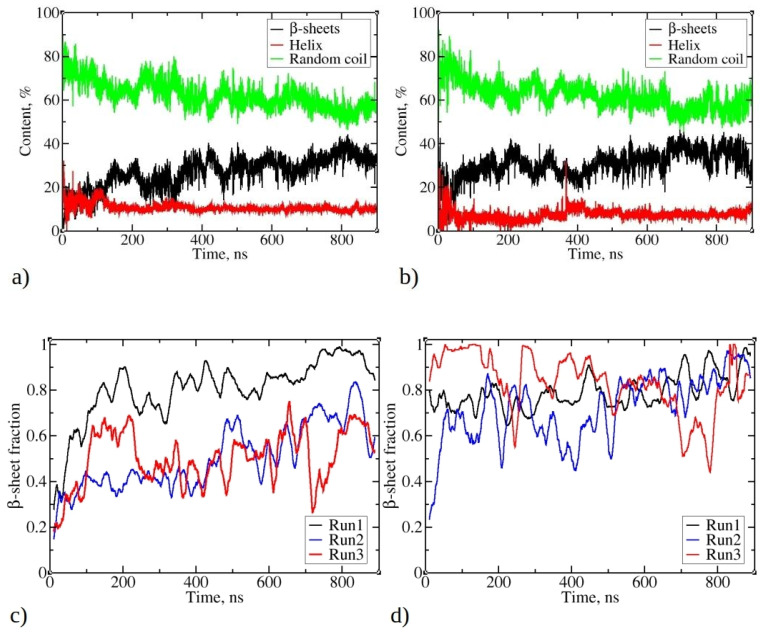
Changes in the total secondary structure in the largest aggregate during simulations of (**a**) 1–33 and (**b**) 1–52 peptides. Total secondary structure is defined as the percentage of residues belonging to all peptides that accordingly adopt β-strands (or are β-sheet-like), helix, or random coil conformation. The fraction of peptide chains of the largest aggregate of (**c**) 1–33 and (**d**) 1–52 that contain β-strands or β-sheets. The β-sheet fraction was calculated as the ratio of peptides, which contain β-strands or β-sheets, divided by cluster size, where 1 means that all peptides in the cluster possess β-strands.

Figure 5a,b illustrates the change of total secondary structure content in the largest aggregates during MD simulations, presented as the percentage of residues with helix, β-strands, or random coil conformations. Notably, β-strands content consistently increases during simulations, resulting in reduced random coil and helix content for both 1–33 and 1–52 peptides. The increase is less noticeable for 1–52, as a portion of their initial structures already includes β-strands (see Figure S3). This is derived from MD snapshots of a single copy of the 1–52 peptide, demonstrating its propensity to fold and adopt a β-strand or β-sheet structure (refer to Figure S2). For 1–33 peptide simulations, β-strands content begins at nearly 0% and gradually rises during the initial 100 ns, fluctuating between 25% and 35% thereafter (Figure 4a). In contrast, helix content decreases from an initial 25% to 10%, remaining stable throughout the simulation. Random coil content decreases from the initial 80% to 60%. Similar trends are observed in 1–52 peptide simulations (as shown in Figure 5b), with β-strands content increasing to values slightly over 40%, while random coil content decreases from 75% to 60%. A slight decrease in a helix content from 12% to 8% via structural transition around 50 ns takes place. 

An in-depth analysis of aggregate characteristics revealed a clear connection between peptide aggregation and the emergence of β-strands and β-sheet structures. Figure 5c,d show a consistent increase in the fraction of peptides adopting β-strands conformations within the largest clusters during the simulation. This fraction varies in different MD runs, ranging from 67% to 95% for simulations involving 1–33 peptides and from 75% to 95% for simulations involving 1–52 peptides. This observation suggests an ongoing transition process, where peptides gradually adopt β-strands conformations, which gradually rearrange in the form of β-sheets. Visual inspection of trajectories shows that β-strands are frequently found in close proximity, exhibiting varying degrees of mutual alignment, akin to β-sheet-like structures. Nevertheless, the β-strands content fluctuates significantly during the simulation, in contrast to the relatively stable sizes of the largest clusters (see Figure 2). This behavior mirrors the extended maturation observed in experimental conditions, where fibril formation typically takes several hours to a day [1].

The fluctuations in β-sheet content during MD trajectories have also been observed in other systems, such as during the formation of steric zipper oligomers [32].

Analyzing the trends in Figure 5a,b, as well as the fraction of peptides adopting β-strand conformations in Figure 5c,d, highlights a notable observation. Even when the fraction of peptides adopting β-strands or β-sheet structures reaches 0.8–0.9, it is evident that the percentage of all residues adopting β-strands (or β-sheet) remains below 40%. This suggests that not all parts of individual peptides have completed the transition into the ordered β-sheet structures.

Data in Figure 5 suggest that, despite observed fluctuations, the largest clusters of 1–52 peptides exhibit slightly higher β-sheet fractions compared to 1–33 peptides. Similar trends are observed for clusters of other sizes, with 1–52 having, on average, higher β-sheet fractions (refer to Figures S6 and S7). For both 1–33 and 1–52 peptides, smaller clusters typically contain a low content of β-strands (Figures S6 and S7), indicating that larger aggregates are necessary to stabilize β-strands. This mechanism contrasts with the aggregation of uperin peptides, where β-strands are already present in dimers, which subsequently merge into larger clusters [33].

**Figure 6:**
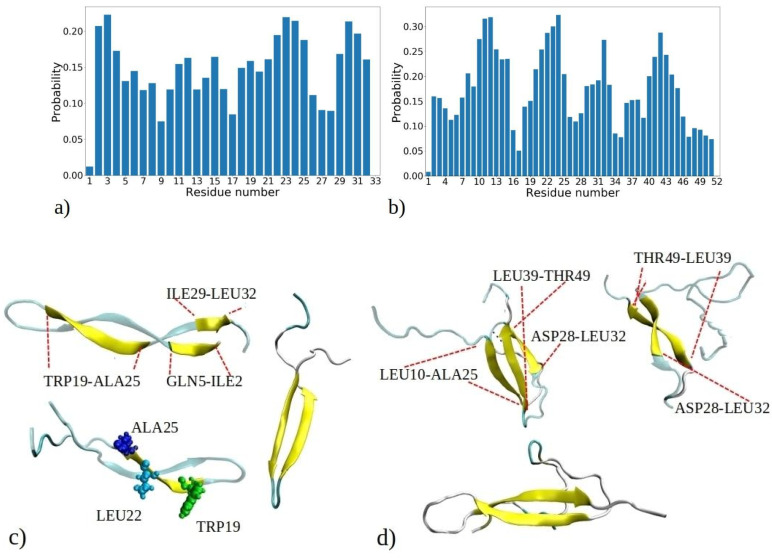
Probability distribution illustrating the propensity of each residue to adopt β-strands or β-sheets as observed in simulations of (**a**) 1–33 and (**b**) 1–52 peptides. The probability is calculated based on the number of events when a residue adopts a β-strand conformation, normalized by the total number of MD snapshots for each of the 20 peptides (i.e., 9000 · 20). Visual snapshots exemplifying the β-sheets structures in (**c**) 1–33 and (**d**) 1–52 peptide chains showcase distinct residues embracing the β-sheets structure (shown by yellow coor).

It is essential not only to examine the total proportion of peptides with β-strands or β-sheet conformations, but also to investigate the intrinsic tendency of individual residues to adopt β-strands conformations within each peptide. These data contribute to a comprehensive atomistic understanding of the formation of fibrils rich with β-sheets. Figure 6 depicts the probability of adopting β-strands or β-sheet conformation of each amino acid residue during the 1–33 and 1–52 MD simulations, determined by counting how often a residue adopts a β-strand conformation compared to the total number of MD snapshots for each of the 20 peptides.

Figure 6a reveals three distinct regions within the 1–33 peptide sequence, which exhibit a strong affinity for β-strand formation: the initial residues (ILE2, VAL3, and THR4), a region around LEU22, ALA23, MET24, and ALA25, and the peptide’s terminus with residues ILE29, SER30, LEU31, and LEU32. The probability of residues with the highest tendency to form β-strands is circa 0.22. Conversely, GLY1, GLY9, and ASP33 have the lowest β-sheet propensity, consistent with glycine’s tendency to avoid β-sheet formation [34]. Similarly, other MD studies have shown that the β2 region of Aβ peptides is less stable compared to the β2 region, attributed to the presence of glycine residues, which facilitate easy movement [35]. These pivotal regions—located at the start, middle, and end of the peptide—act as nucleation sites for initiating β-sheet assembly, influencing other segments to adopt β-strand and β-sheet configurations. Notably, many residues located within regions highly prone to β-strand formation consist of hydrophobic amino acids commonly observed in β-strands of other proteins, such as VAL, ILE, TRP, TYR, THR, and more [34]. Several regions of residues exhibiting a high propensity for β-sheet formation are also observed in MD simulations of the aggregation of amyloid-β (Aβ) and human islet amyloid polypeptide (hIAPP) peptides into homodimers and heterodimers [36]. The MetAmyl webserver [37] identified hotspot regions within the 1–33 peptide sequence, spanning from LEU1 to LYS8 and from ILE12 to GLY17, partially correlating with MD data. 

In the 1–52 peptides, a region with heightened β-sheet propensity emerges between residues 39 and 46, particularly in ARG40, VAL41, TYR42, VAL43, and GLU44 (Figure 6b). Notably, 1–52 showcases a distinct region with lower β-sheet frequency, spanning residues 34 to 39. Among them is proline (PRO38), which is known for disrupting β-strands due to its inability to complete hydrogen bonding networks [34,38]. The behavior of the first 33 residues in 1–52 peptides reflects a similar trend to that of 1–33 peptides, with some distinctions, including expanded regions prone to β-sheet formation, such as contiguous sequence of residues 8 to 15. Clearly, the environment created by larger 1–52 peptides increases the likelihood of these residues forming β-strands compared to 1–33 peptides. Thus, the β-sheet propensity of each residue arises from both its inherent tendency to adopt β-strands and the influence of neighboring residues in its vicinity. According to predictions made by the MetaAmyl webserver [37], the hotspot regions of the 1–52 peptide span from LEU1 to LYS8, from ILE12 to GLY17, and from PRO38 to GLU44. In the case of 1–52 peptides, residues with the highest tendency to form β-strands have slightly higher probability than 1–33 peptides, i.e., circa 0.32. This aligns with MD simulations of a single 1–52 peptide, where the peptide chain exhibited increased folding and formed intramolecular β-sheets (refer to Figure S2). Additionally, it corresponds to the data in Figure S6, indicating that aggregates of 1–52 peptides have higher fractions of β-sheets. The visual snapshots in Figure 6c,d illustrate the adoption of distinct β-strand or β-sheet structures by both 1–33 and 1–52 peptides. Demonstrating a predominantly folded configuration, the peptides exhibit a tendency for intramolecular β-sheet formation, which can then establish further intermolecular contacts with β-sheet of adjacent chains. U-shaped conformations characterized by intramolecular β-strands, similar to those observed in snapshots from MD, have also been reported for Aβ and hIAPP peptides in other studies [35,39]. The observed probabilities of each residue adopting β-sheets for both the 1–33 and 1–52 peptides correlate with predictions from the PASTA 2.0 webserver [40], except for the first five residues of 1–33 and 1–52, as well as the 39 to 46 region of 1–52, which exhibit higher probabilities according to PASTA 2.0 compared to the MD data (Figure S12). These discrepancies can be attributed to limitations of each model, and limited number of available amyloid sequences on which the PASTA 2.0 model was tuned.

### 2.4. Hydrogen Bonds and Cluster Orderliness 

**Figure 7:**
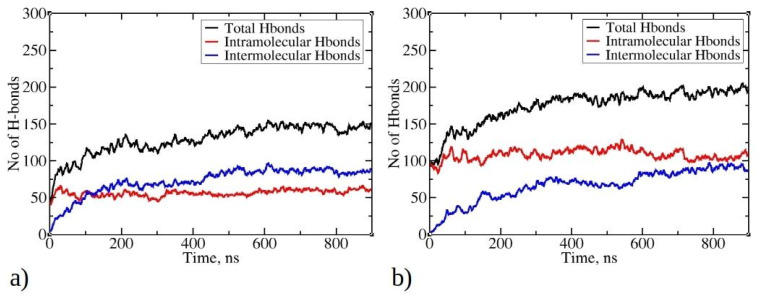
Time evolution of total, intermolecular, and intramolecular hydrogen bonds during MD simulations for (**a**) 1–33 and (**b**) 1–52 peptides. Only data from Run1 are presented for each peptide, as main trends remained consistent across multiple runs.

Hydrogen bonds play a crucial role in stabilizing protein systems and peptide aggregates [41,42]. In Figure 7, the time evolution of total, intermolecular, and intramolecular hydrogen bonds during MD simulations is illustrated. As the simulation progresses and peptides undergo aggregation, the total number of hydrogen bonds increases, starting with an initial count of 50 for 1–33 peptides and 100 for 1–52 peptides. By the end of the simulation, there are 200 hydrogen bonds for 1–52 peptides and 150 for 1–33 peptides. Notably, the larger size of 1–52 peptides enables them to establish more bonds than their 1–33 counterparts. As the aggregation process of peptides is initiated, they commence the formation of intermolecular hydrogen bonds with other peptides within clusters (aggregates). The most rapid increase in intermolecular hydrogen bonds is observed within the first 200 ns for 1–33 peptides simulations and within the initial 300 ns for 1–52 peptides simulations, coinciding with the formation of larger aggregates (see Figure 2). Subsequently, the increase in intermolecular hydrogen bonds continues, albeit at a slower pace, indicating a gradual structural rearrangement within the aggregates. This process reaches 88 intermolecular hydrogen bonds for 1–33 peptides and 93 for 1–52 peptides at the end of the simulations. Examining the relationship between the radius of gyration of peptides and the number of intermolecular hydrogen bonds per contact suggests that hydrogen bonds are formed concomitantly with aggregation (Figure S8). The data do not exhibit the typical L-shape characteristic of the initial hydrophobic collapse preceding the establishment of native contacts [43,44]. Interestingly, the number of intramolecular hydrogen bonds does not undergo significant changes during simulations of both peptide types, except for minor fluctuations. This is likely due to the predominance of folded conformations adopted by the peptides, facilitating the establishment of intramolecular interactions. For larger 1–52 peptides, the number of intramolecular hydrogen bonds exceeds that of intermolecular hydrogen bonds (Figure 7b). This is likely one of the contributing factors to the formation of smaller clusters for 1–52 peptides (see Figure 2b), as aggregation typically involves conformational rearrangements and the establishment of new intermolecular interactions. Visual inspection of trajectories reveals the formation of hydrogen bonds between β-strands, within helices, and in random coil regions (Refer to Figure S9 for visual snapshots).

For 1–33 peptides, hydrogen bonds commonly occur between residues such as SER30-ILE2, TYR20-TYR20, SER21-GLN5, and so on (Refer to Figure S10a for the contact map of intermolecular hydrogen bonds). In addition to hydrogen bonding, TRP19 and TYR20 residues may also engage in π–π stacking interactions, introducing stabilizing factors for aggregates. Work by Porat et. al. underscores the significance of aromatic residues such as TRP19 and TYR20 in stabilizing amyloid aggregates due to their stacking interactions [45]. Since the aggregates formed during the MD runs represent early assembly intermediates that have not yet matured into well-ordered fibrils, hydrogen bond formation is largely influenced by the local environment and the dynamic rearrangement of peptide chains. The most frequently occurring intramolecular hydrogen bonds, such as THR18-LYS14 and SER27-ALA23, are predominantly found in helices (See Figure S11a). For 1–52 peptides, common hydrogen bonds form between PRO50-VAL3, LYZ14-LEU32, MET7-TYR20, and so on (Figure S10b). TYR20 frequently engages in hydrogen bonding with various residues, aligning with the observed pattern in 1–33 peptides. Similarly, like 1–33 peptides, intramolecular hydrogen bonds in the 1–52 system, such as ASP33-ILE29 and LEU32-ASP28 (Figure S11b), persist due to long lasting helical motifs during simulations.

**Figure 8:**
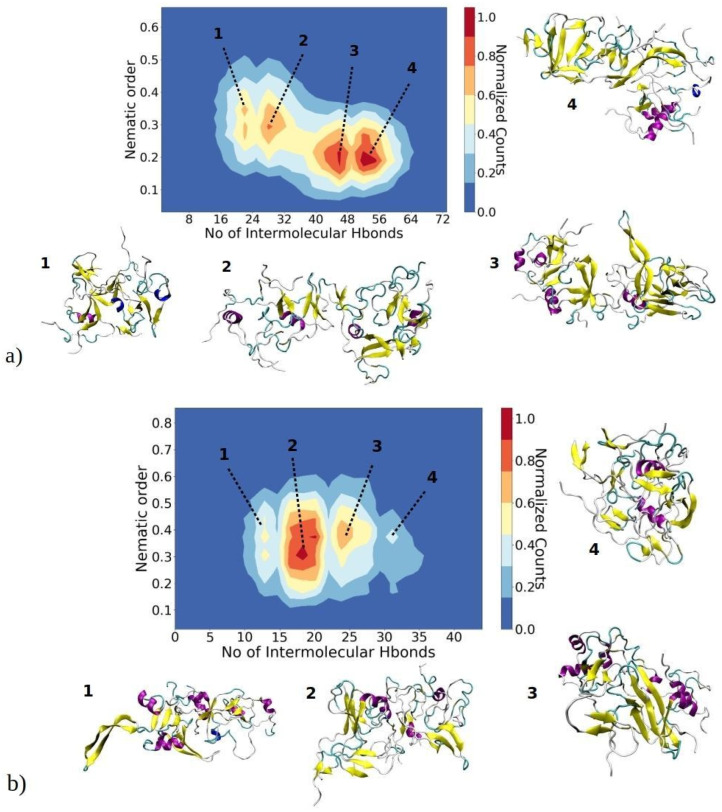
A 2D contour plot illustrates the relationship between the aggregate nematic order parameter and the number of inter-chain hydrogen bonds for the largest aggregates of (**a**) 1–33 and (**b**) 1–52 peptides. The *x*-axis represents the number of inter-peptide hydrogen bonds, while the *y*-axis depicts the nematic order parameter of the aggregate. The color contours represent the normalized counts of data points, with a transition from blue to red. Peptides in aggregates belonging to basins 1, 2, 3 or 4 are color-coded to represent their secondary structure: yellow for β-sheets, purple for helices, and cyan for random coils.

As amyloid fibrils are frequently stabilized by hydrogen bonds between adjacent β-strands, our investigation delved into the relationship between the orderliness of aggregates and the number of intermolecular hydrogen bonds formed within each aggregate, as depicted in Figure 8. This offers valuable insights into the correlation between molecular interactions, quantified by hydrogen bonds, and the orderliness of the largest aggregates, determined by their nematic order parameter. These two parameters serve to describe (pre-)fibrillization states of aggregates, as a higher content of β-sheets leads to a larger nematic order parameter, and the more perfectly aligned β-sheets should enable the formation of a larger number of hydrogen bonds. The nematic order parameter for the largest aggregates of 1–33 peptides ranges between 0.15 and 0.55, whereas for 1–52 peptides, it falls within the range of 0.15 to 0.65. This observation suggests that these aggregates are in a pre-fibrillar stage, as fibrils typically exhibit an order parameter greater than 0.8 [43,46]. The 2D contour plots for 1–33 peptides reveal the presence of four distinct regions, each representing a predominant basin where the majority of observed structures are concentrated (Figure 8a). In the first basin, the nematic order parameter ranges from 0.25 to 0.32, while the number of intermolecular hydrogen bonds falls between 18 and 22. Visual snapshots indicate that structures belonging to this basin exhibit β-strands without parallel ordering. Structures in basin 2, characterized by a similar range of nematic order parameters but a larger number of hydrogen bonds, display a higher content of beta strands that are more ordered and arranged in close proximity to each other. In basins 3 and 4, structures exhibit a slightly lower order parameter (approximately 0.2); yet, they showcase a higher number of intermolecular hydrogen bonds—around 42 to 46 and 50 to 56, respectively. Representative visual snapshots clearly reveal that these aggregates contain a larger quantity of β-strands, though their arrangement is not uniform throughout the entire structure. This localized arrangement contributes to the observed lower order parameter.

Similarly, structures of the largest aggregates of 1–52 peptides concentrate in four different basins (Figure 8b). In the first basin, structures exhibit an order parameter in the range of 0.35 to 0.45, while the number of intermolecular hydrogen bonds is relatively low (around 10–13). Consequently, clusters feature poorly aligned β-strands with a significant portion of helical and random coil structures. As the number of intermolecular hydrogen bonds increases, clusters tend to adopt more ordered β-strands, as seen in basins 2 and 3. Visual snapshots show that these aggregates feature several regions of nearly parallelly aligned β-strands; however, there is still no long-range alignment. Thus, clusters in these basins have an order parameter in the range of 0.28 to 0.5. It can be assumed that over a prolonged time, these β-strands would perfectly align concomitant with cluster growth, reaching a critical nucleus size. The slightly more ordered 1–52 aggregates can most likely be attributed to the longer peptide chain, which contains additional hydrophobic residues (Figure 1c). These additional hydrophobic residues create an environment conducive to additional β-sheet stabilization.

Finally, it is crucial to recognize that the concentrations of peptides in our MD simulations exceeded typical experimental concentrations due to computational constraints. Experimental evidence suggests that higher concentrations of BLG lead to aggregates with reduced β-sheet content [11]. Additionally, the possibility exists that the critical nucleus size for fibrillization is higher than the number of peptides simulated, acting as a limiting factor in achieving structural arrangement into β sheets. Furthermore, the considerable size and high flexibility of these peptides may contribute to the prolonged time required for fibril formation in experiments. Bellesia et al. employed an off-lattice coarse-grained peptide model to investigate the influence of β-sheet propensity on the structural properties of peptide aggregates [46]. Through systematic adjustments to both β-sheet propensity and peptide rigidity, the study unveiled that increased peptide flexibility triggers a transition from fibrillar structures to a diverse array of formations, including β-barrels and, ultimately, completely amorphous aggregates. The notably high flexibility of BLG peptides simulated in this study provides insight into the structural diversity observed in aggregates, as well as the experimentally observed slow kinetics of fibril assembly. 

## 3. Materials and Methods

The initial structures for the 1–33 and 1–52 peptides were extracted from the crystal structure of the BLG protein, obtained from the Protein Data Bank with the 3npo code [47]. The peptide structures were protonated at pH 2 using the PROPKA method (version 3.3) [48,49] and the PDB2PQR online web server [50], resulting in charges of +3 and +5 for 1–33 and 1–52 peptides, respectively.

MD simulations of solutions comprising 1–33 or 1–52 BLG peptides were conducted with GROMACS package (version 2019.2) [51,52] and were modeled using the CHARMM36m force field [53]. Water was described using the CHARMM36m modified TIP3 potential [54], allowing increased protein–water interactions and preventing over-stabilization of protein structure. Previous comparative studies have shown that the CHARMM36m force field, with a modified water potential [53,54], yields more promising results for peptide simulations compared to other state-of-the-art force fields [55,56]. The H-O bonds and H-O-H angles were constrained using the LINCS algorithm [57]. To maintain charge neutrality, 10 mM NaCl, typically used in experiments [1], was added and also modeled with CHARMM36m parameters. The simulations employed a timestep of 2 fs, with short-range nonbonded interactions cutoff at 1.2 nm. Full electrostatic interactions beyond 1.2 nm were computed using the Particle Mesh Ewald (PME) algorithm [58]. 

Initially, MD simulations of a single 1–33 or 1–52 peptide were conducted to generate various starting conformations, which were further utilized for MD simulations involving twenty peptide chains in one simulation box. Each peptide was placed in a periodic cubic box measuring 6.4 × 6.4 × 6.4 nm^3^. The system underwent a 5000-step minimization using the steepest descent algorithm, with position restraints applied to the heavy atoms of the proteins. Subsequently, it was equilibrated for 600 ps under canonical (NVT) conditions at 353.15 K (i.e., 80 °C, which is used in experiment for peptide assembly [1]), while restraining the protein atoms. A Nosé−Hoover thermostat was employed [59], with a temperature damping parameter of 100 fs. Finally, a 500 ns unrestrained production run for each of the peptides was conducted under isothermal–isobaric conditions (NPT) using the Parrinello–Rahman barostat [60] at 353.15 K and 1 bar, with a pressure damping parameter of 1000 fs and a temperature damping factor of 100 fs. A total of 20 random peptide conformations were selected from the trajectory. These random conformations served as initial configurations for simulations with 20 peptides. 

For these simulations, 20 copies of 1–33 or 1–52 peptides were placed in a periodic cubic box measuring 15 × 15 × 15 nm^3^ and 16.5 × 16.5 × 16.5 nm^3^, respectively, corresponding to a macroscopic concentration of 9.303 mmol/L and 7.6 mmol/L. Twenty peptides were chosen as a compromise between computational efficiency and feasibility to observe larger aggregates, which are expected to be more representative of early-stage aggregates and to more efficiently stabilize secondary structure changes. The systems were simulated using MD setup in a manner consistent with the single peptide simulations. Production runs with the duration of 900 ns each were conducted three times for each peptide system. Trajectory snapshots were saved every 100 ps, and subsequent analysis of MD trajectories was conducted using GROMACS tools or custom scripts with the MDanalysis Python library [61,62]. Peptide aggregate (cluster) size distributions over MD were determined by custom script using the head and list algorithm, which assigns molecules to clusters based on a chosen distance cutoff of 4 Å between atoms of different peptides. In this algorithm, three vectors (CLUSTER, HEAD, and LIST) with a dimension of 20 (representing the total number of peptides in the simulation box) are utilized to store the indices of molecules belonging to the same cluster and their respective cluster identities [63,64]. For convenience, a fourth vector (SIZE) is introduced to store the size of each cluster. Initially, all molecules are considered to belong to individual single-molecule clusters, with CLUSTER and HEAD storing their respective indices, while LIST is initialized as 0. The algorithm iterates through pairs of molecules/peptides (i and j), assigning two molecules to the same cluster if their distance is below a chosen cutoff and they do not already belong to the same cluster. Subsequently, HEAD (i) is updated to point to molecule j, LIST (j) is set to i, and HEAD [j] is set to 0. The elements of the CLUSTER array for molecules i and j are then updated to share the same cluster ID, and the SIZE vector is adjusted accordingly. Upon completion of the algorithm, nonzero values in HEAD indicate the number of clusters identified, with each nonzero HEAD (i) corresponding to the remaining molecules/peptides in the cluster, as stored in LIST. Consequently, molecules belonging to the same cluster can be identified by traversing the LIST array starting from each nonzero HEAD (i) = j, followed by LIST (j) = k, LIST (k) = l, and so forth, until reaching a zero-valued cell in LIST.

Secondary structure was determined by using the VMD program [65]. Hydrogen bonds were identified using a custom script with the MDanalysis library. The criteria for recognizing hydrogen bonds included a distance cutoff of 1.2 Å for donor–hydrogen distance, a donor–acceptor distance cutoff of 3.0 Å, and a donor–hydrogen–acceptor angle cutoff of 150 degrees. The MetAmyl webserver (http://metamyl.genouest.org/e107_plugins/metamyl_aggregation/db_prediction_meta.php accessed on 12 April 2024) [37] was employed to identify hotspot regions within peptide sequences that serve as initiation sites for amyloid aggregation, while the PASTA 2.0 webserver (http://old.protein.bio.unipd.it/pasta2/index.html accessed on 12 April 2024 [40] was utilized to predict the secondary structure propensity of individual residues. Both of these computational methods utilized FASTA peptide sequences as input.

## 4. Conclusions

In this study, molecular dynamics simulations were employed to investigate the aggregation behavior of N-terminal 1–33 and 1–52 BLG peptides at pH 2 and a 10 mM NaCl concentration, chosen due to their significance in BLG fibril formation under prolonged heating. While previous theoretical studies have predominantly focused on amyloid proteins with shorter sequences and greater rigidity, our work sheds light on the aggregation behavior of longer, more flexible BLG peptides. To ensure robust statistical analysis, three independent MD runs were conducted for each peptide type. Through these simulations, peptides spontaneously assembled into aggregates of varying sizes, with the largest aggregates reaching twelve, thirteen, and ten peptides for 1–33 peptides, and six, seven, and six for 1–52 peptides. Through careful comparison with other molecular dynamics studies in the field of amyloid aggregation, we have elucidated unique structural and kinetic characteristics of BLG aggregation. The aggregation process was aided by the low peptide charge and the presence of hydrophobic residues, which are common factors enabling amyloid aggregation. This was further supported by the consistent decrease in SASA observed during MD runs, where hydrophobic residues aggregated to minimize contact with water. Initially forming small- and intermediate-sized clusters with random coil conformations, peptides progressed in simulations to adopt β-sheet structures. Approximately 10% and 8% of residues in 1–33 and 1–52 peptides, respectively, assumed helical structures persisting throughout simulations. While 1–33 peptides formed larger aggregates on average, 1–52 peptides exhibited a slightly higher β-sheet content. Detailed analysis of secondary structure changes revealed specific regions in both peptide types prone to β-sheet formation, acting as nucleation points for the progression to more ordered structures. Aggregation was accompanied by the concurrent establishment of intermolecular and intramolecular hydrogen bonds between peptides, further stabilizing the aggregates. A higher count of intramolecular hydrogen bonds corresponds to a greater degree of order in aggregates, wherein the regions of aggregates display parallel alignment of β-strands. In summary, this initial MD study sheds light on the early steps of BLG peptide assembly and the molecular mechanism of the nucleation phase preceding ordered fibril growth. Our findings underscore the importance of considering peptide length and flexibility in understanding amyloid formation, and highlight the potential utility of our methodological approach in studying diverse amyloidogenic proteins and peptides. This study marks the first theoretical prediction of BLG peptide early aggregate structures without prior knowledge of experimentally determined structures. Moreover, it lays the groundwork for future investigations aiming to enhance our understanding of BLG fibril structure through collaborative efforts with experimental research. The insights garnered from MD simulations can inform the design of novel peptide sequences with tailored properties. Through targeted residue substitutions, peptide structures can be engineered to enhance or diminish β-sheet propensity and kinetics. Techniques such as accelerated MD or free energy methods can be employed to investigate the effects of mutations on peptide structure and stability. The experimental structure of these aggregates remains to be revealed, potentially opening new avenues of research.

## Data Availability

The datasets generated during the current study are available from the corresponding author on reasonable request. The input files for MD runs, along with the initial and final snapshots of each MD simulation, are available in the NOMAD repository accessible via the provided https://doi.org/10.17172/NOMAD/2024.04.24-2 link (accessed on 12 April 2024).

## Supplementary Materials

The following supporting information can be downloaded at https://www.mdpi.com/article/10.3390/ijms25094660/s1.
