# Supplementary material for: Molecular Dynamics Insights into the Aggregation Behavior of N-Terminal β-Lactoglobulin Peptides
Source: Int J Mol Sci. 2024 Apr 25;25(9):4660. doi: 10.3390/ijms25094660 (PMC11083573; doi:10.3390/ijms25094660)
Supplement: Supplementary file 1 [file ijms-25-04660-s001.zip › ijms-2952210-supplementary.pdf]

# Molecular Dynamics Insights into the Aggregation Behavior of N-terminal β-lactoglobulin Peptides

Srdjan Pusara

Institute of Nanotechnology, Karlsruhe Institute of Technology KIT, Kaiserstraße 12, 76131 Karlsruhe, Germany

Corresponding author, srdjan.pusara@partner.kit.edu

# Supporting Information

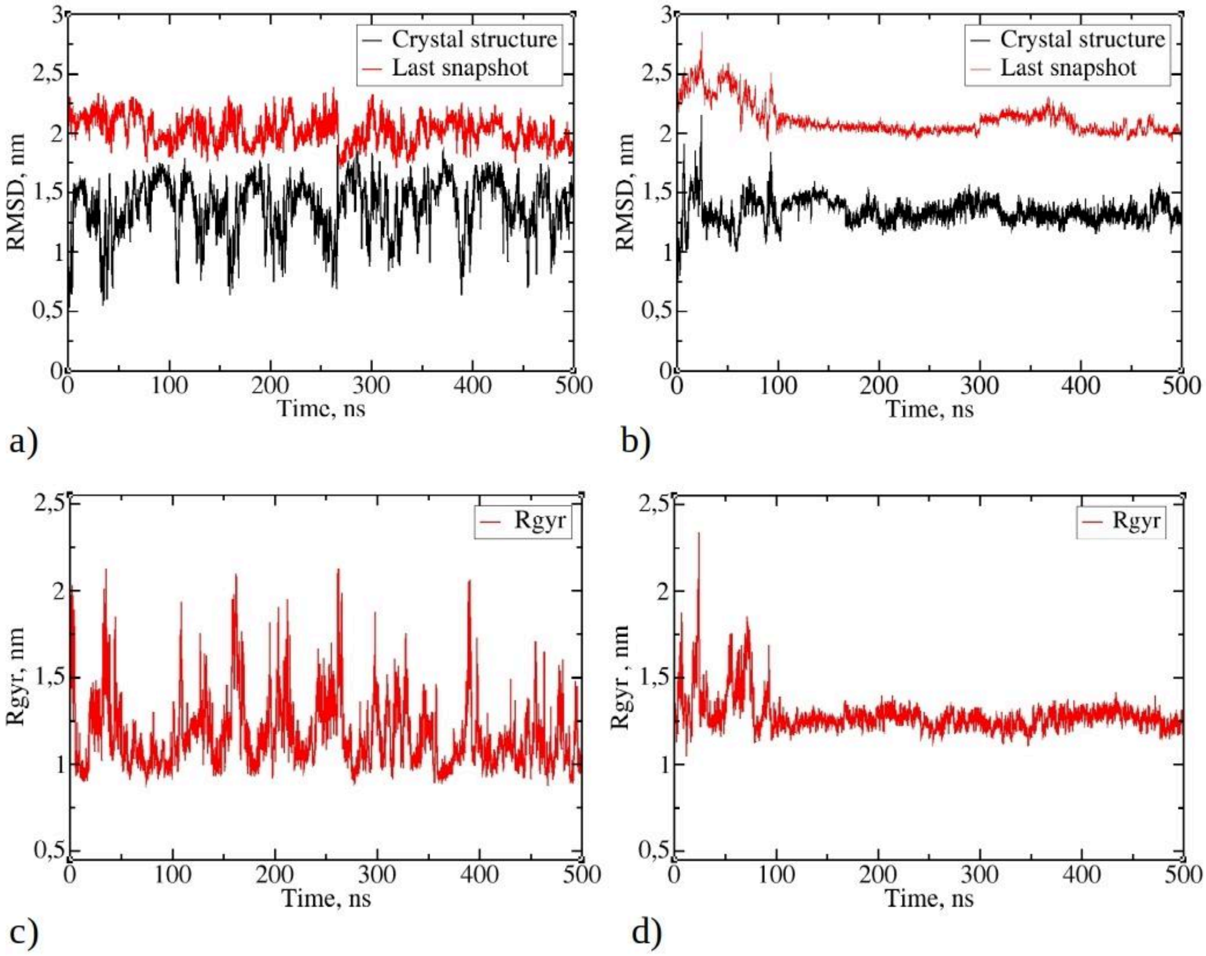

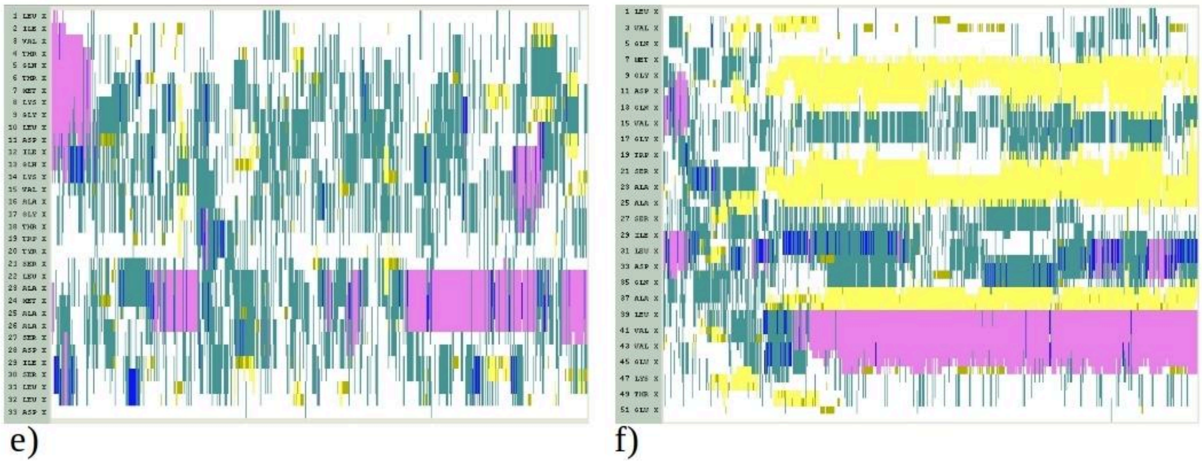


**Figure S1.** Change of root mean square deviation (RMSD) during MD simulations of single copy of a) 1-33 and b) 1-52 peptide. The reference structure for calculating RMSD was either the peptide structure extracted from the PDB crystal structure or the last snapshot of the MD simulation. Change of radius of gyration of (Rgyr) during MD simulations of single copy of c) 1-33 and d) 1-52 peptides. Change of secondary structure during MD simulations of single copy of e) 1-33 and f) 1-52 peptides. β-strands, α-helix, 3-10 helix and random coils are marked by yellow, purple, blue and cyan color, respectively.

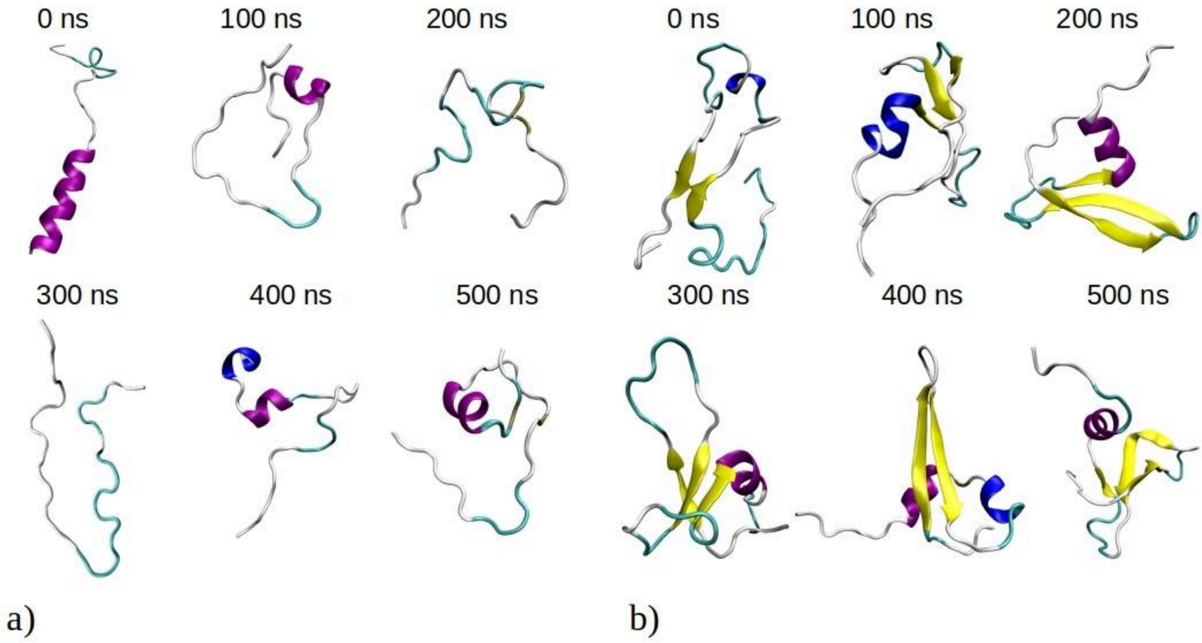


**Figure S2.** Visual snapshots from MD simulations of single copy of a) 1-33 and b) 1-52 peptide. For each simulation snapshot is shown at t=0 ns, t= 100 ns, t=200 ns, t=300 ns, t=400ns and t=500 ns. β-strands, α-helix, 3-10 helix and random coils are marked by yellow, purple, blue and cyan color, respectively.

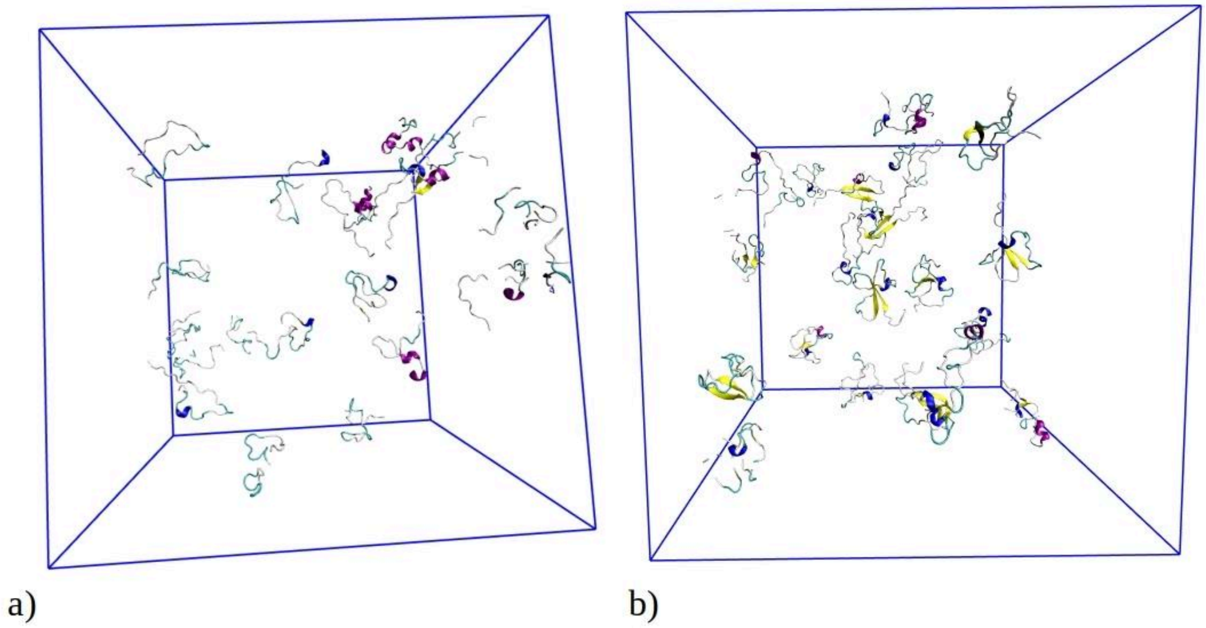


**Figure S3.** Visual snapshots of starting configurations for MD simulations of twenty copies of a) 1-33 (Run1) and b) 1-52 (Run1) peptide. β-strands, α-helix, 3-10 helix and random coils are marked by yellow, purple, blue and cyan color, respectively.

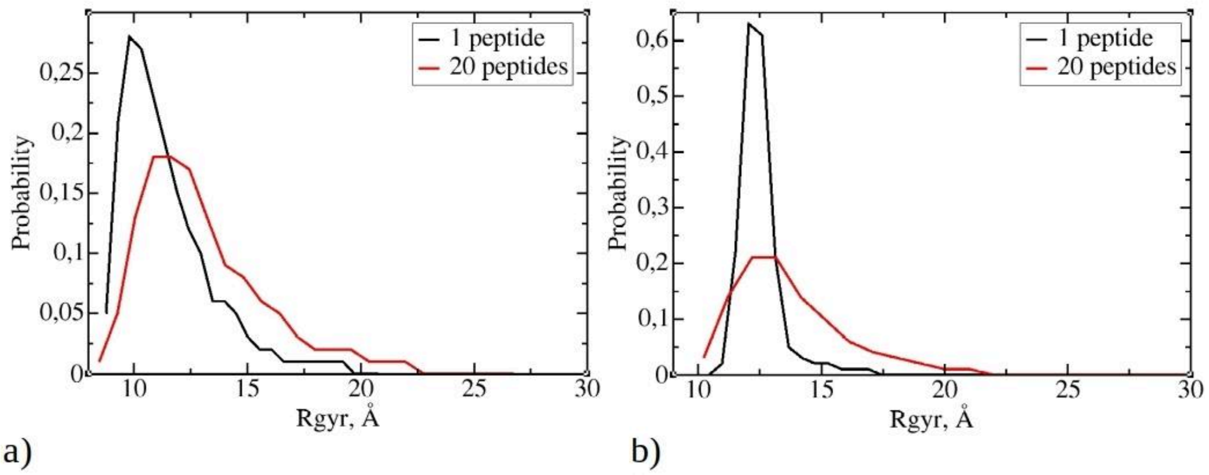


**Figure S4.** Histogram of radius gyration (Rgyr) distribution in MD simulations of single copy and twenty copies of a) 1-33 and b) 1-52 peptides.

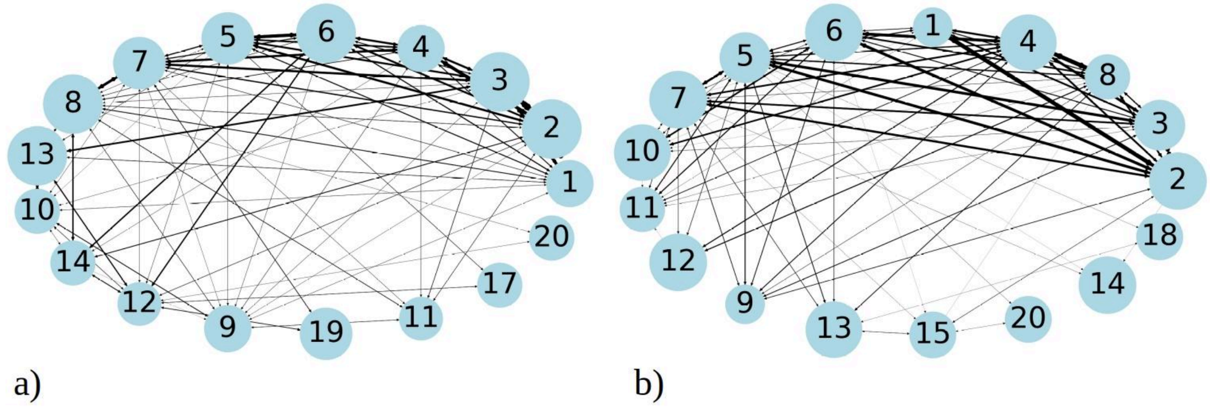

**Figure S5.** Transition matrix illustrating all observed transitions between cluster sizes for a) 1-33 and b) 1-52 peptide simulations. The data presented in these plots include statistics from all three MD runs combined, for 1-33 and 1-52 peptides respectively. The size of the circle is proportional to the total number of transitions from/into that state, while the thickness of the line is proportional to the number of events between two states.

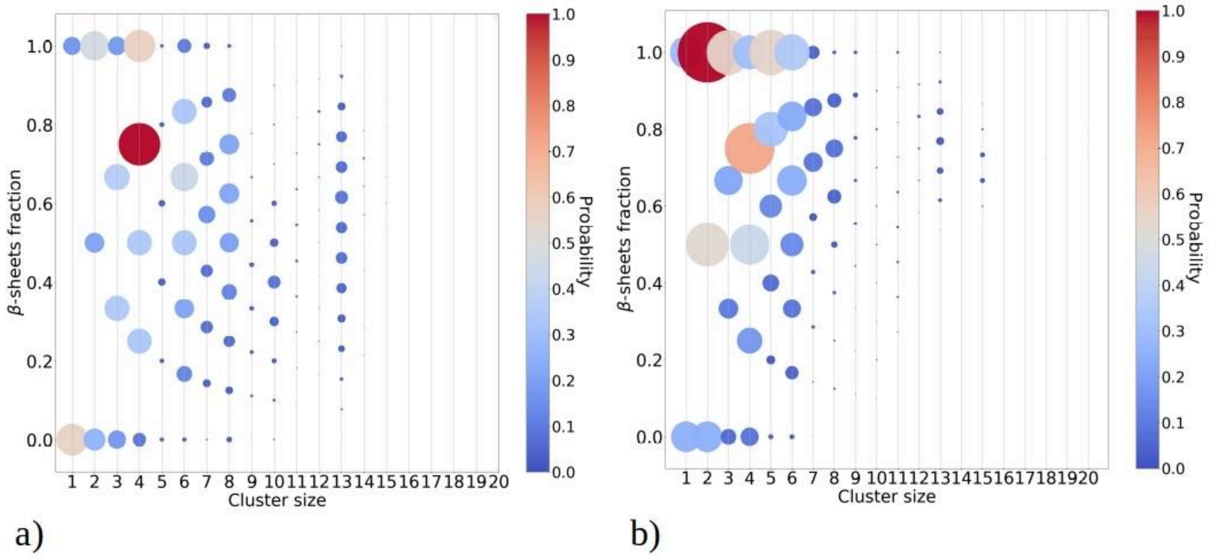


**Figure S6.** Scatter plot illustrating the relationship between cluster size (x-axis) and β-sheet fraction (y-axis) for observed aggregates (clusters) during MD simulations of a) 1-33 and b) 1-52 peptides. The β-sheet fraction was calculated as the ratio of peptides which contain β-strands or β-sheets divided by cluster size, where 1 means that all peptides in the cluster possess β-strands.

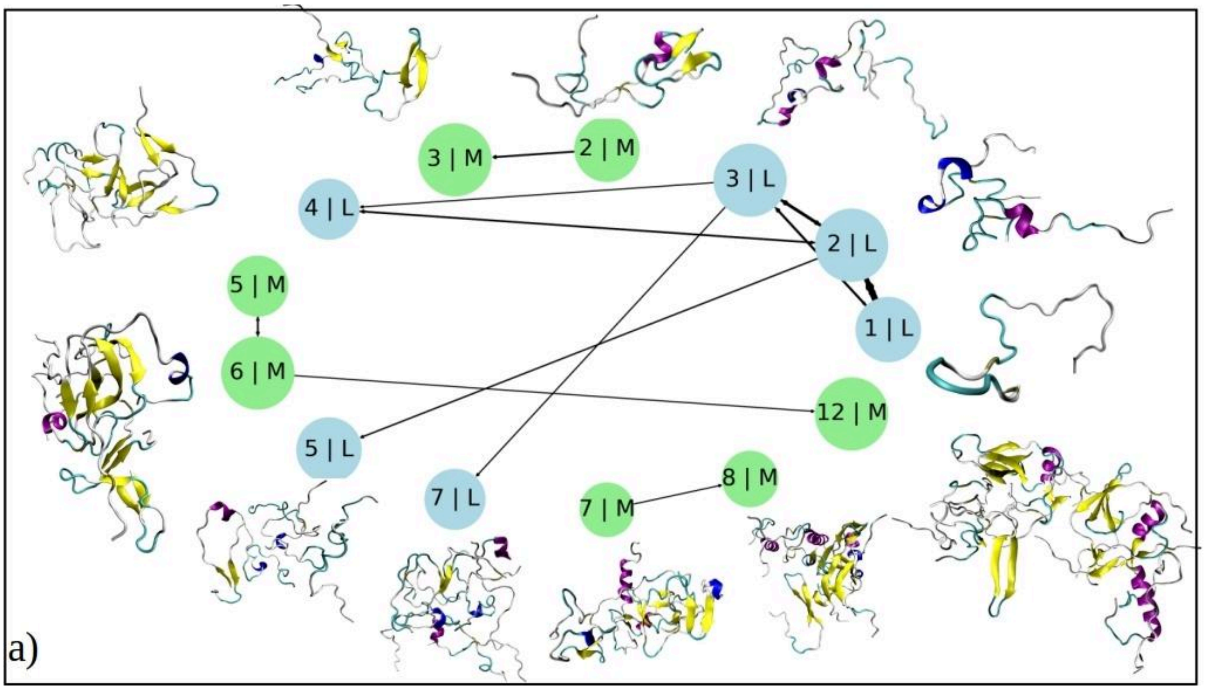

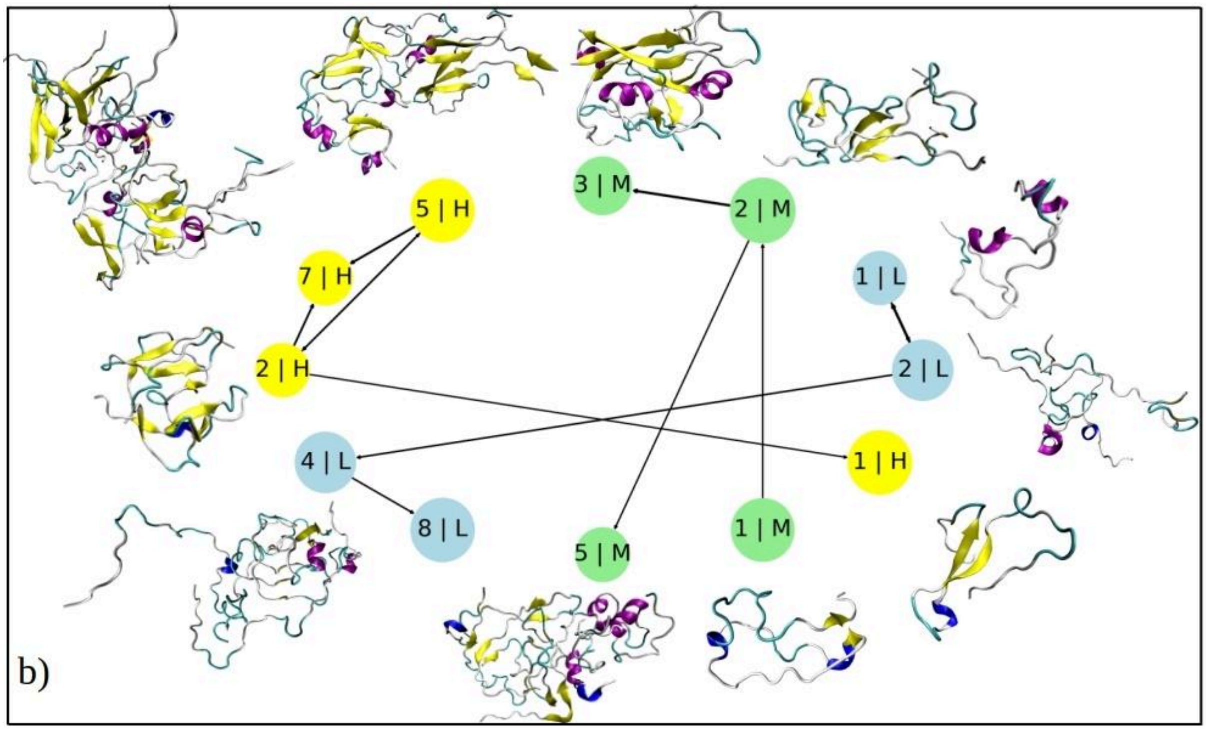


**Figure S7.** Transition networks graph which illustrates the 16 most frequent transitions between aggregate states observed during simulations of a) 1-33 and b) 1-52 peptides. Aggregate states are defined as a combination of the number of peptides which are found within the cluster, and the fraction of peptides with β-strands (or β-sheets) structure, denoted as ‘L’, ‘M’ and ‘H’. The fraction of β-strands content was calculated as the ratio of peptides adopting β-strands conformation versus total cluster size. Here ‘L’, ‘M’ and ‘H’ denote that the total fraction of peptides falls <0.3, between 0.3 and 0.7 and >0.7, respectively.

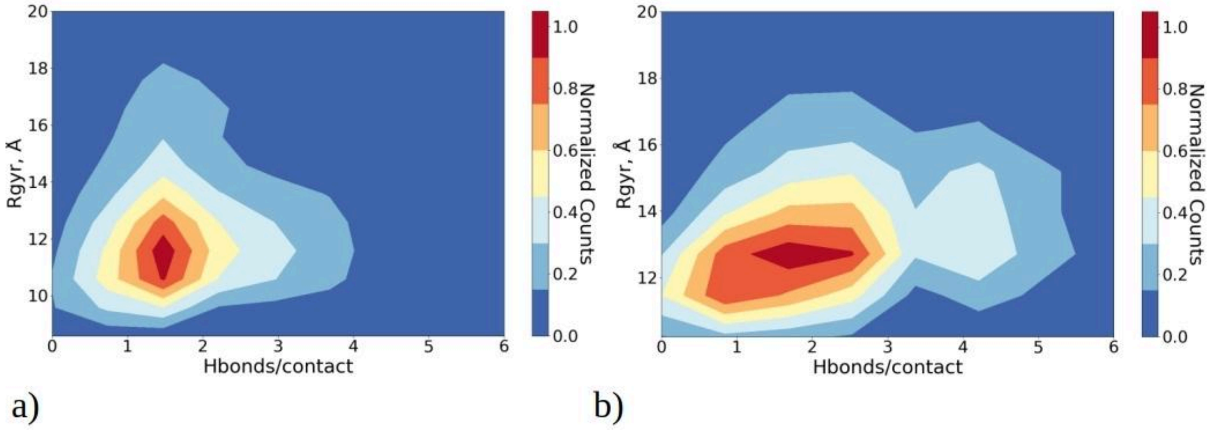


**Figure S8.** A 2D contour plot illustrates the relationship between the Rgyr of peptide and the number of inter-chain hydrogen bonds during MD simulations of a) 20 copies of 1-33 peptides and b) 20 copies of 1-52 peptides. The x-axis represents the number of inter-peptide hydrogen bonds (normalized by the number of contacts of each peptide), while the y-axis depicts the Rgyr of the peptide. The color contours represent the normalized counts of data points, transitioning from blue to red.

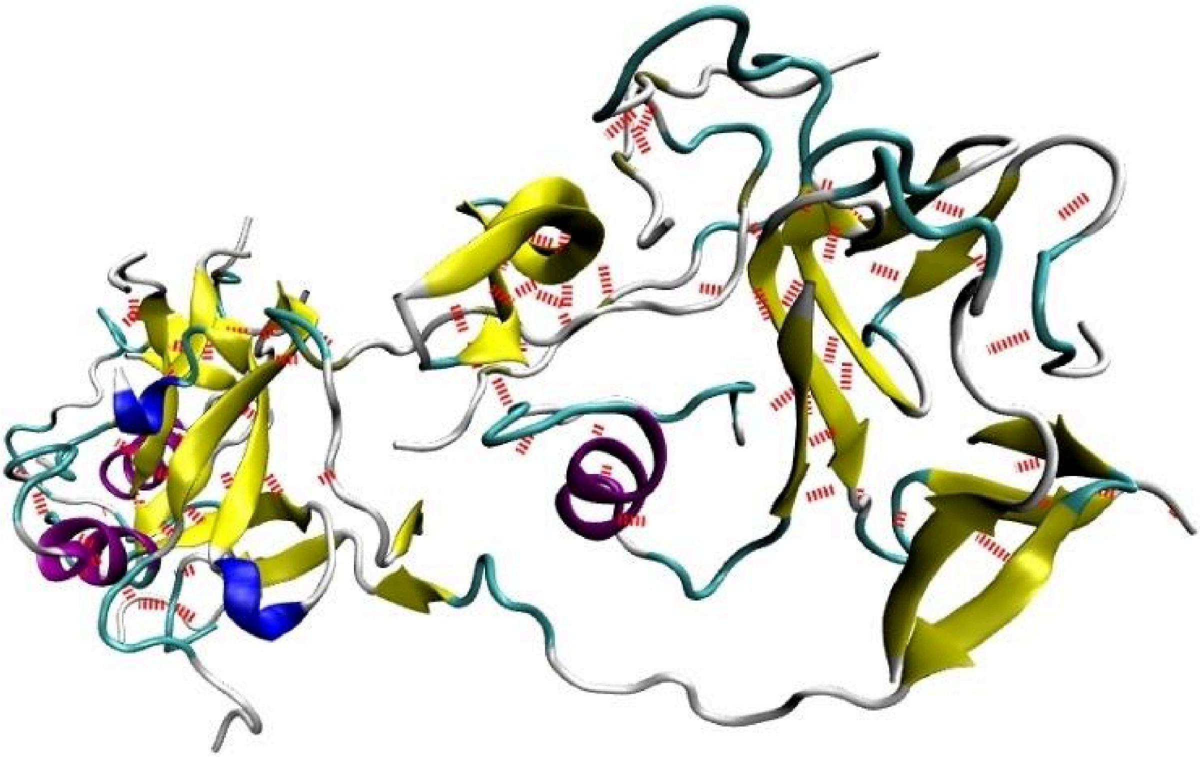

a)

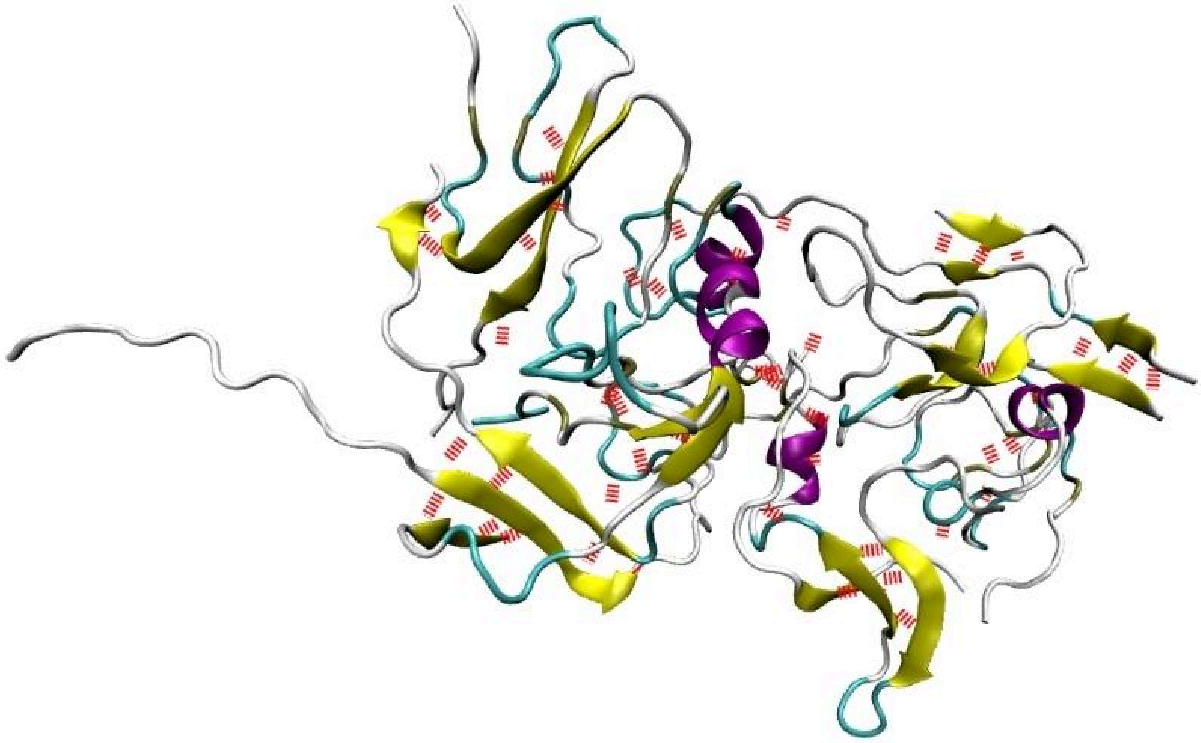

b)

**Figure S9.** Visual snapshots depict the hydrogen bonding network within the largest clusters formed during MD simulations of 20 copies of a) 1-33 peptides and b) 1-52 peptides. In the

illustrations, β-strands, α-helices, 3-10 helices, and random coils are denoted by yellow, purple, blue, and cyan colors, respectively. Hydrogen bonds are depicted by red dashed lines. The illustrated cluster of 1-33 consists of 13 peptides, whereas the cluster of 1-52 comprises 7 peptides. Hydrogen bonds are depicted by dashed red lines.

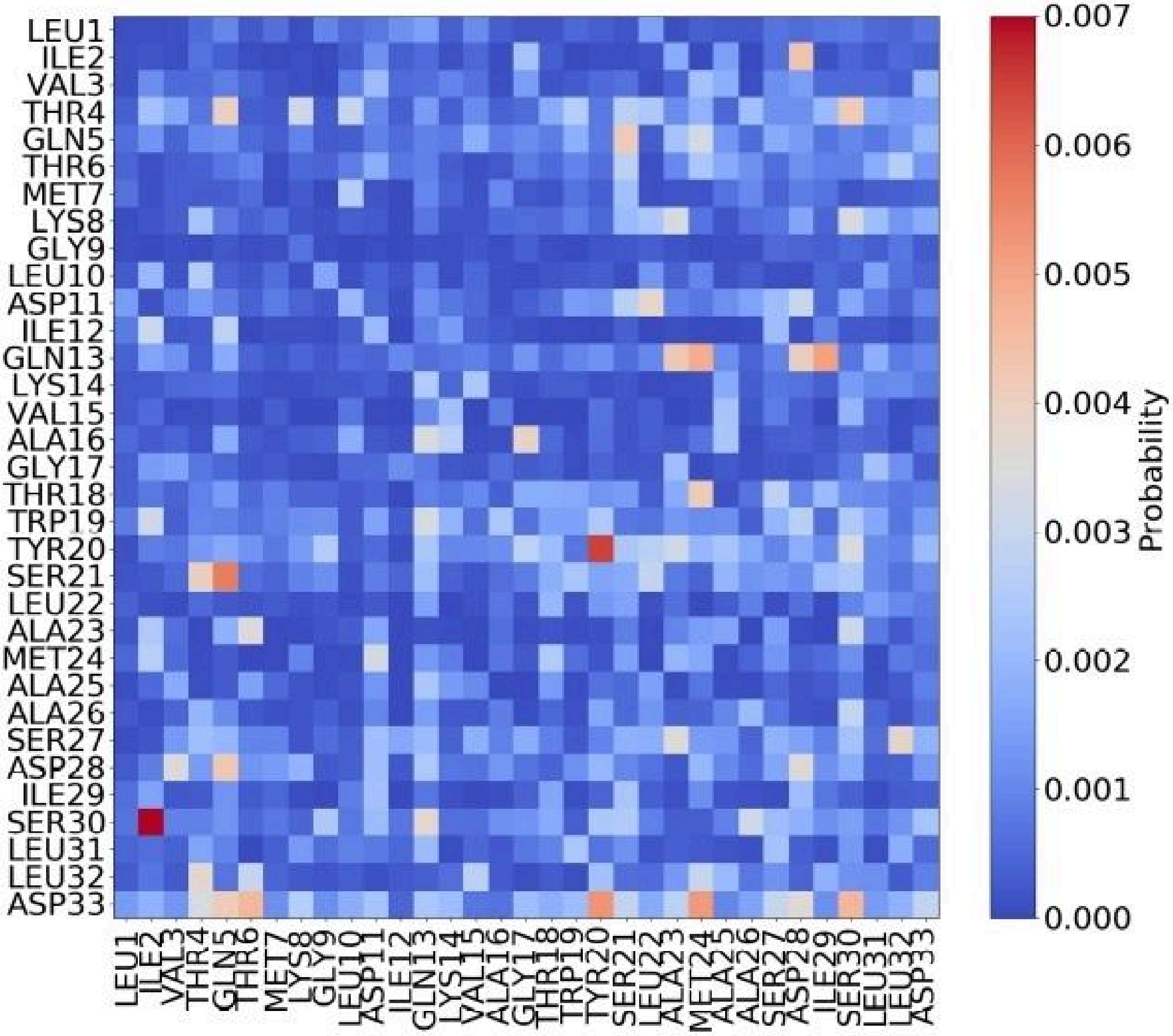


a)

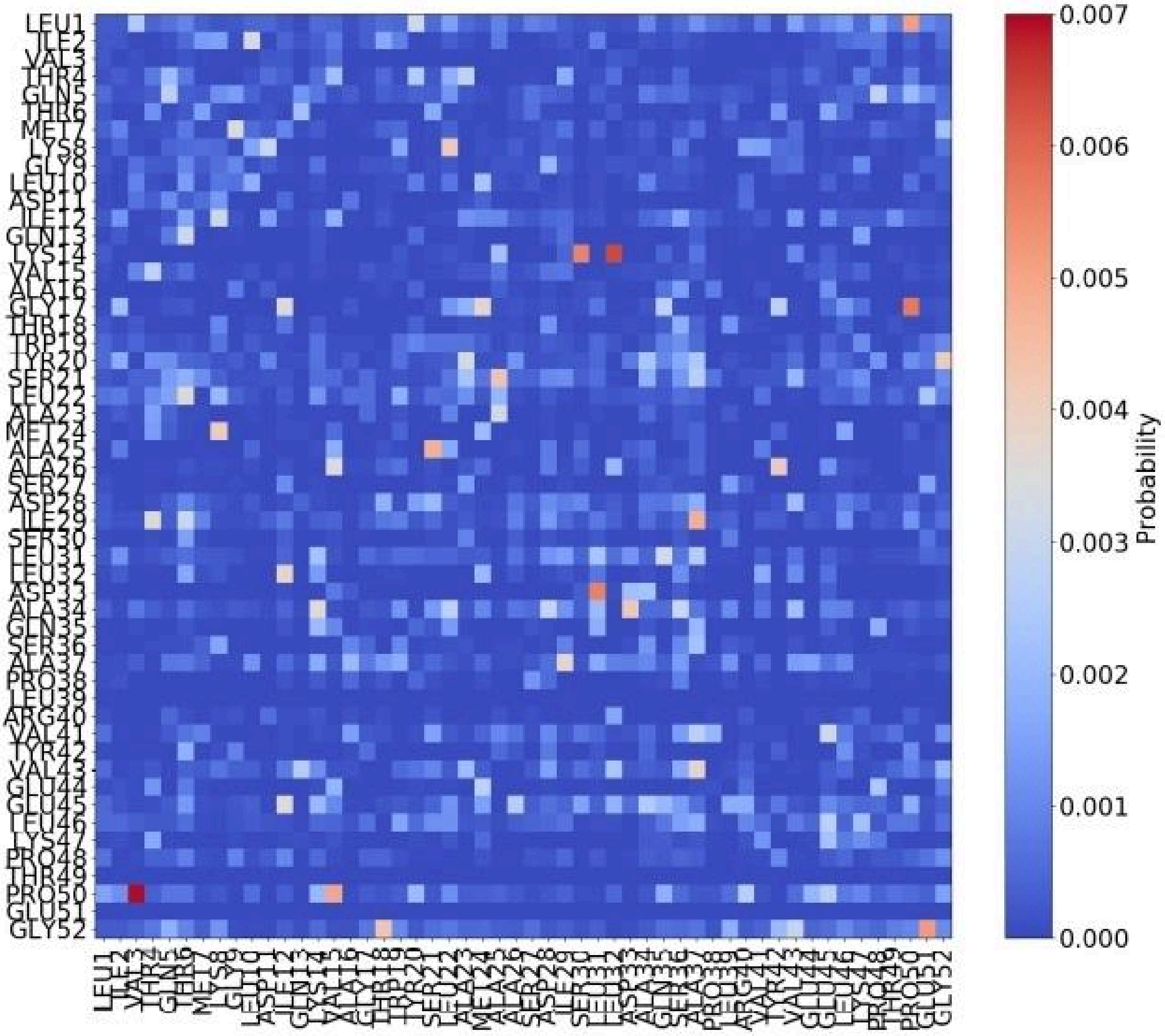


b)

**Figure S10.** Contact map illustrating the frequency of occurrence of intermolecular hydrogen bonds between different residues during MD simulations of a) 20 copies of 1-33 peptides and b) 20 copies of 1-52 peptides. The data are normalized based on the total number of counts.

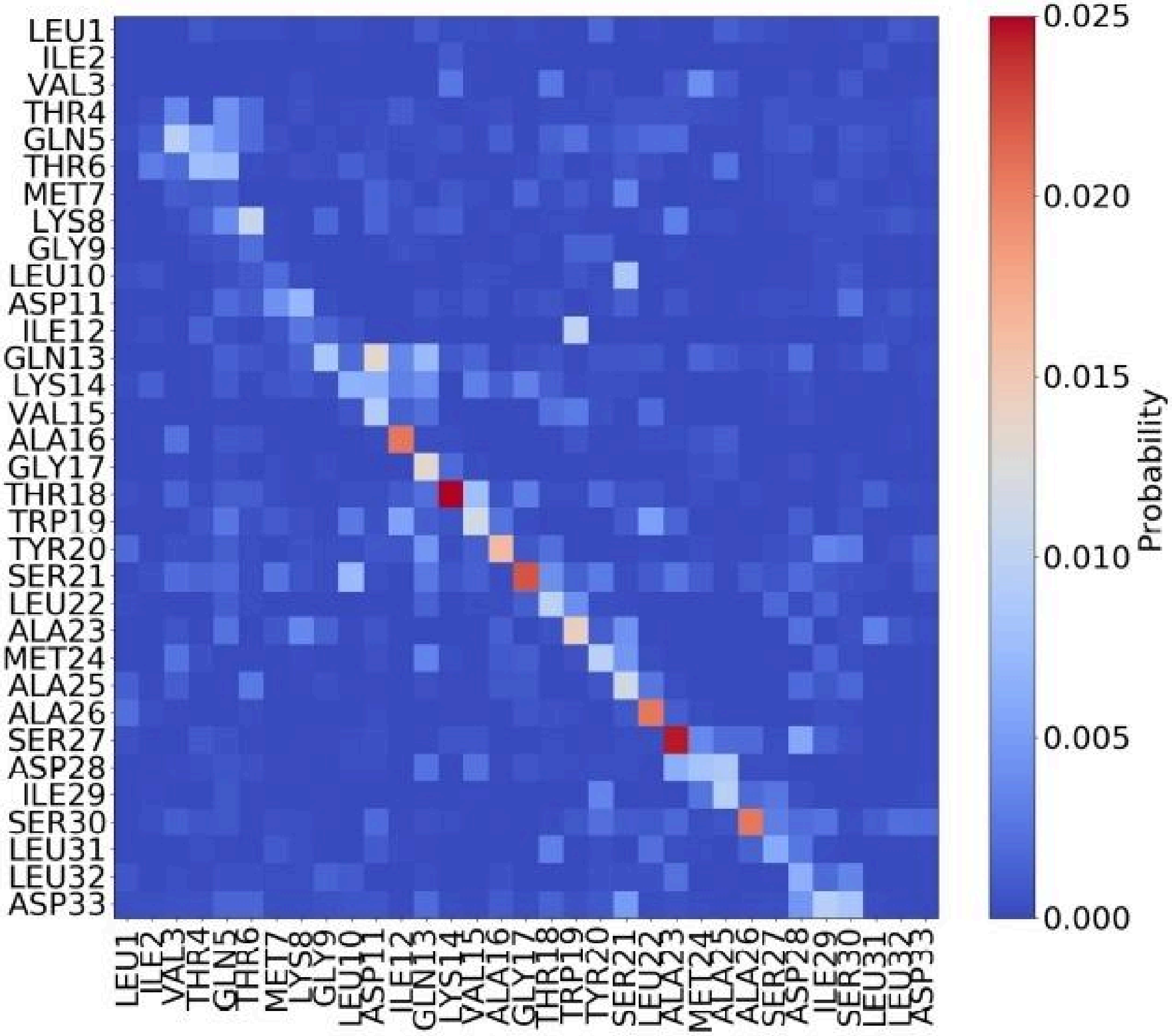

LEU1
ILE2
VAL3
THR4
GLN5
THR6
MET7
LYS8
GLY9
LEU10
ASP11
ILE12
GLN13
LYS14
VAL15
ALA16
GLY17
THR18
TRP19
TYR20
SER21
LEU22
ALA23
MET24
ALA25
ALA26
SER27
ASP28
ILE29
SER30
LEU31
LEU32
ASP33
0.025
0.020
0.015
0.010
0.005
0.000
Probability


a)

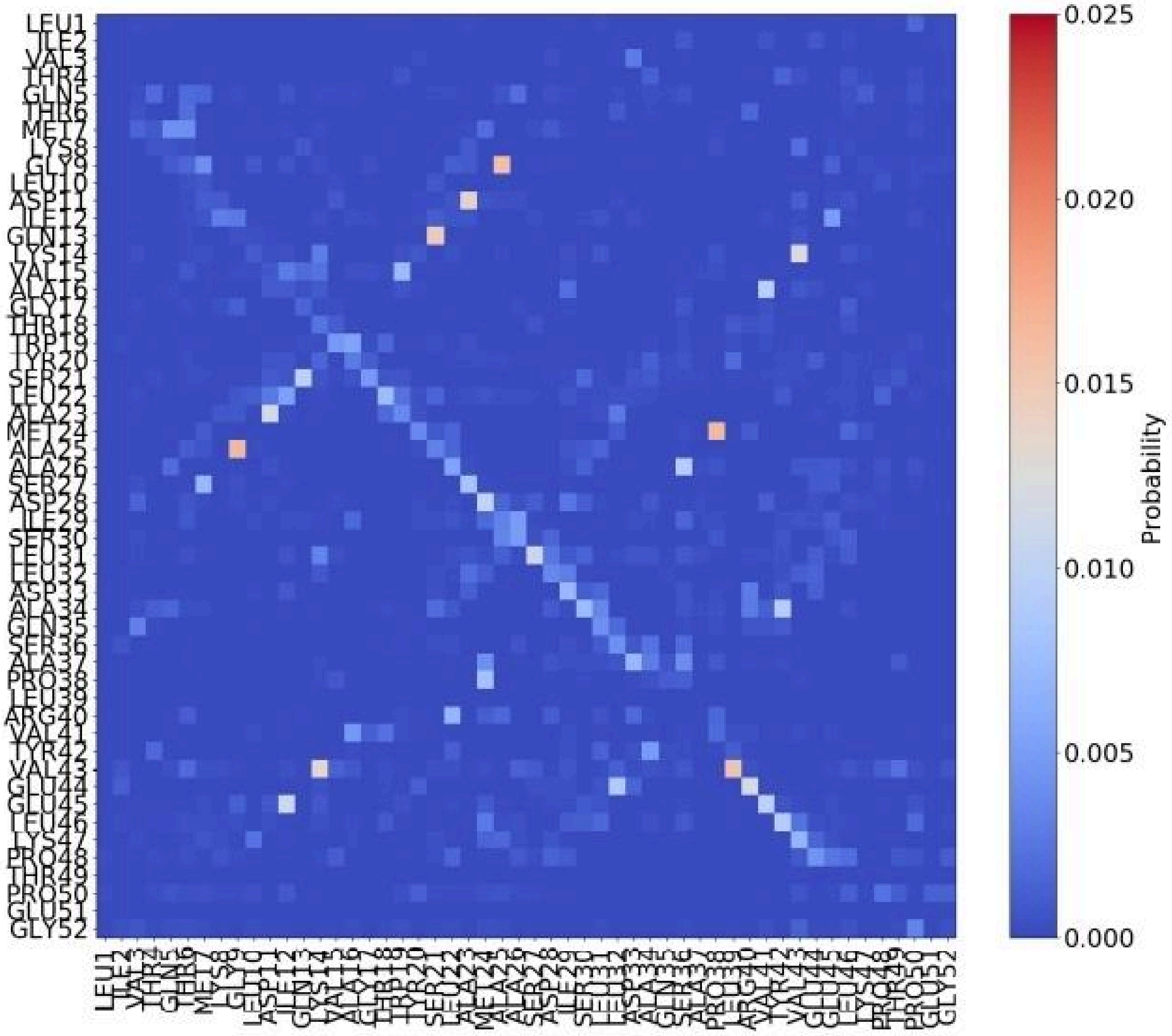


b)

**Figure S11.** Contact map illustrating the frequency of occurrence of intramolecular hydrogen bonds between different residues during MD simulations of a) 20 copies of 1-33 peptides and b) 20 copies of 1-52 peptides. The data are normalized based on the total number of counts.

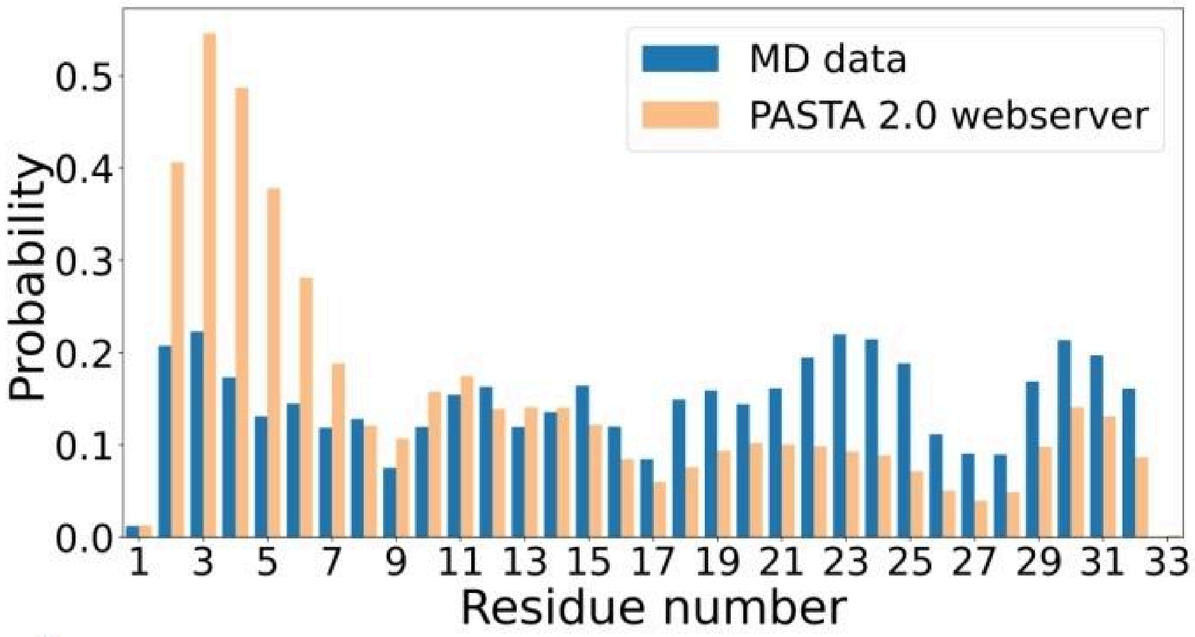


a)

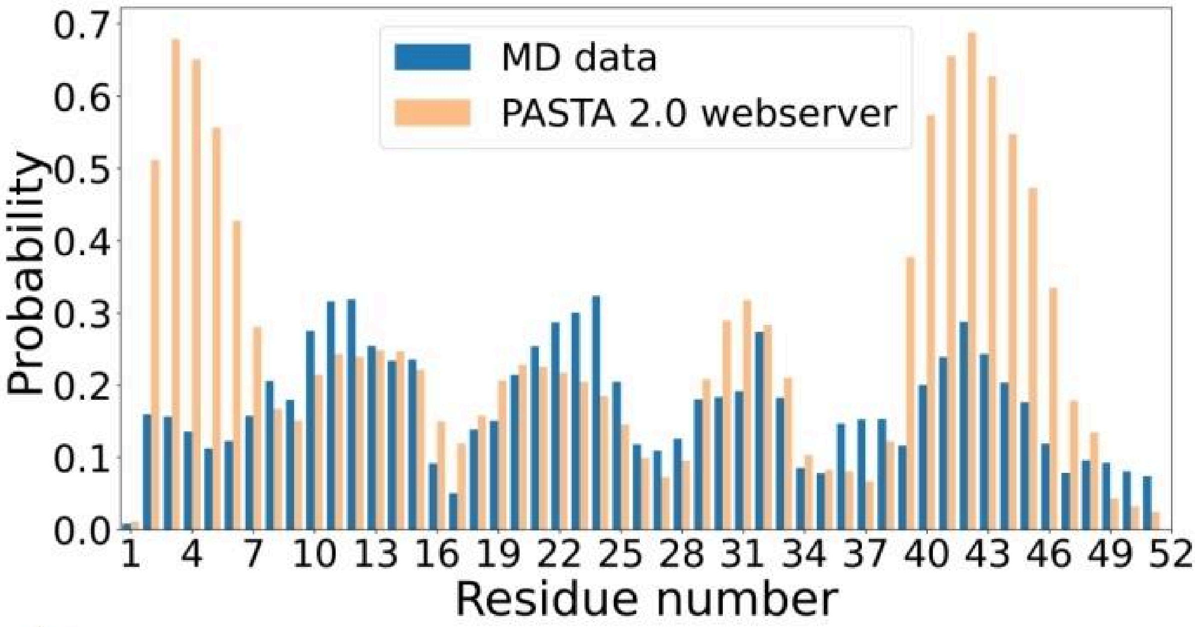


a)

**Figure S12.** Probability distribution depicting the propensity of each residue to adopt β-strands or β-sheets, as determined from MD simulations and by the PASTA 2.0 webserver for a) 1-33 and b) 1-52 peptides. The MD-derived probability is calculated based on the frequency of occurrences where a residue adopts a β-strand conformation, normalized by the total number of MD snapshots for each of the 20 peptides (i.e., 9000 × 20).
